# Feasibility and effects of intra-dialytic low-frequency electrical muscle stimulation and cycle training: A pilot randomized controlled trial

**DOI:** 10.1371/journal.pone.0200354

**Published:** 2018-07-11

**Authors:** Gordon McGregor, Stuart Ennis, Richard Powell, Thomas Hamborg, Neil T. Raymond, William Owen, Nicolas Aldridge, Gail Evans, Josie Goodby, Sue Hewins, Prithwish Banerjee, Nithya S. Krishnan, Stephen M. S. Ting, Daniel Zehnder

**Affiliations:** 1 Health & Life Sciences Faculty Research Centre, Coventry University, Coventry, United Kingdom; 2 Department of Nephrology, University Hospital, Coventry, United Kingdom; 3 Cardiff Centre for Exercise & Health, Cardiff Metropolitan University, Cardiff, United Kingdom; 4 Department of Cardiac Rehabilitation, Centre for Exercise & Health, University Hospital, Coventry, United Kingdom; 5 Statistics and Epidemiology, Division of Health Sciences, Warwick Medical School, University of Warwick, Warwick, United Kingdom; 6 Department of Cardiology, University Hospital, Coventry, United Kingdom; 7 Warwick Medical School, University of Warwick, Warwick, United Kingdom; 8 Department of Acute Medicine, North Cumbria University Hospital NHS Trust, Carlisle, United Kingdom; Fondazione Toscana Gabriele Monasterio, ITALY

## Abstract

**Background and objectives:**

Exercise capacity is reduced in chronic kidney failure (CKF). Intra-dialytic cycling is beneficial, but comorbidity and fatigue can prevent this type of training. Low–frequency electrical muscle stimulation (LF-EMS) of the quadriceps and hamstrings elicits a cardiovascular training stimulus and may be a suitable alternative. The main objectives of this trial were to assess the feasibility and efficacy of intra-dialytic LF-EMS vs. cycling

**Design, setting, participants, and measurements:**

Assessor blind, parallel group, randomized controlled pilot study with sixty-four stable patients on maintenance hemodialysis. Participants were randomized to 10 weeks of 1) intra-dialytic cycling, 2) intra-dialytic LF-EMS, or 3) non-exercise control. Exercise was performed for up to one hour three times per week. Cycling workload was set at 40–60% oxygen uptake (VO_2_) reserve, and LF-EMS at maximum tolerable intensity. The control group did not complete any intra-dialytic exercise. Feasibility of intra-dialytic LF-EMS and cycling was the primary outcome, assessed by monitoring recruitment, retention and tolerability. At baseline and 10 weeks, secondary outcomes including cardio-respiratory reserve, muscle strength, and cardio-arterial structure and function were assessed.

**Results:**

Fifty-one (of 64 randomized) participants completed the study (LF-EMS = 17 [77%], cycling = 16 [80%], control = 18 [82%]). Intra-dialytic LF-EMS and cycling were feasible and well tolerated (9% and 5% intolerance respectively, *P* = 0.9). At 10-weeks, cardio-respiratory reserve (VO_2 peak_) (Difference vs. control: LF-EMS +2.0 [95% CI, 0.3 to 3.7] ml.kg^-1^.min^-1^, *P* = 0.02, and cycling +3.0 [95% CI, 1.2 to 4.7] ml.kg^-1^.min^-1^, *P* = 0.001) and leg strength (Difference vs. control: LF-EMS, +94 [95% CI, 35.6 to 152.3] N, *P* = 0.002 and cycling, +65.1 [95% CI, 6.4 to 123.8] N, *P* = 0.002) were improved. Arterial structure and function were unaffected.

**Conclusions:**

Ten weeks of intra-dialytic LF-EMS or cycling improved cardio-respiratory reserve and muscular strength. For patients who are unable or unwilling to cycle during dialysis, LF-EMS is a feasible alternative.

## Introduction

The severely reduced exercise capacity associated with chronic kidney failure (CKF) is an inevitable consequence of hypertension, chronic uraemia and low grade systemic inflammation. With maintenance hemodialysis, functional ability is further compromised due to fatigue and inactivity [1]. Structural and functional changes to the cardiovascular system and skeletal muscle contribute to an exercise capacity (peak oxygen uptake, VO_2 peak_) that is commonly only 50–60% of normal [2, 3]. Consequently, activities of daily living are curtailed and quality of life can be poor [4–6].

The progressive effects of CKF are evident throughout the cardiovascular system. Increased vascular resistance, endothelial dysfunction and fibrotic left ventricular hypertrophy lead to a marked increase in cardiovascular disease (CVD) mortality and disease specific morbidity [7, 8]. Equally debilitating is the inflammatory cytokine and inactivity mediated imbalance in protein homeostasis which results in the catabolic destruction of structural and functional proteins with skeletal muscle wasting [9]. Medical strategies to combat these ill effects are limited. However, some of the cardiovascular and skeletal muscle sequelae that typify the CKF phenotype are modified by exercise interventions in other disease states [10]. As such, there may be scope for structured exercise training during hemodialysis to attenuate CKF specific cardiovascular dysfunction.

Cardiovascular exercise, most commonly cycle training, has been shown to be well tolerated during hemodialysis [11]. In small trials, numerous benefits have been reported including improved oxygen uptake, muscular strength, arterial compliance and inflammation [2, 12, 13]. Moreover, physical activity between dialysis sessions is improved, as is quality of life and other measures of psychosocial functioning [14]. For some, however, dynamic exercise is not possible due to orthopaedic limitations, unstable hemodynamics during dialysis, or simply low motivation, malaise and fatigue. It is imperative, therefore, that alternative exercise modalities are considered, to allow incapacitated patients to benefit from cardiovascular exercise training.

Low-frequency electrical muscle stimulation (LF-EMS) of the quadriceps and hamstrings can evoke rhythmical sub-tetanic muscle activation and an acute cardiovascular response similar to that of dynamic exercise [15]. In chronic heart failure exercise capacity can be improved, dyspnoea reduced and health related quality of life (HR-QoL) enhanced [16–19]. Intra-dialytic LF-EMS may be a suitable alternative to dynamic exercise training when the latter is unachievable. In a previous exploratory study, comparable improvements in strength, six-minute walk distance and HR-QoL were reported in patients completing dynamic intra-dialytic exercise or a tetanic LF-EMS protocol [20]. To address the paucity of data in this area, and to establish the feasibility and potential therapeutic benefits of this intervention, it is important to conduct further trials using LF-EMS during hemodialysis.

In patients with CKF, the main objective of this pilot study was to compare the feasibility of LF-EMS and dynamic cycle training during hemodialysis. We also aimed to investigate the effects of intra-dialytic LF-EMS and cycle training, compared to usual care, on exercise capacity and cardiovascular structure and function.

## Materials and methods

### Participants

Patients receiving hemodialysis at a tertiary centre and satellite units were screened for eligibility. Inclusion was dependent upon age >18 years, dialysis three times weekly for 3–4 hours, dialysis vintage of >3 months, urea reduction rate of >65%, and ability to complete dynamic exercise testing and training. Exclusion criteria were active malignant disease, ischemic cardiac event (< 3 months), significant valvular heart disease or dysrhythmia, planned kidney transplant during the study period, and life expectancy of <6 months. Data was collected between March 2014 and September 2015.

### Study procedure

The protocol was approved by the West Midlands Research Ethics Committee (13/WM/0494, 23^rd^ December 2013) and registered with ClinicalTrials.gov: NCT02874521 (retrospectively due to an administrative error relating to online institutional approval). The authors confirm that there are no ongoing or related trials for this intervention. Participants provided written informed consent and the study adhered to the declaration of Helsinki. Baseline procedures were conducted in a single visit on a non-dialysis day, in advance of randomization. Demographics, standard clinical parameters and a full medical history were recorded before proceeding with blood sampling, echocardiography, arterial applanation tonometry, flow mediated dilation, leg strength dynamometry and cardiopulmonary exercise testing (CPET). Permuted block randomization (block sizes 3 and 6) was stratified by sex and age (55 years) and performed independently by the trial statistician. Participants were allocated to 10 weeks of 1) intra-dialytic low-frequency electrical muscle stimulation (LF-EMS), 2) intra-dialytic cycle training, or 3) usual care with no exercise training. Post study measures were conducted on a non-dialysis day by experienced outcome assessors blinded to group allocation. As the primary outcome was the assessment of feasibility, no power calculation was conducted.

### Primary outcome: Feasibility

The feasibility of intra-dialytic LF-EMS and cycle training was measured in several ways. Firstly, recruitment rates were recorded to determine the willingness of participants to engage in LF-EMS and cycling. Secondly, retention and intervention tolerability were established by monitoring study drop-out rates and reasons. Finally, the number of exercise sessions completed in accordance with the protocol was taken as a measure of adherence.

### Secondary outcomes

#### Cardio-respiratory reserve

Maximal CPET was performed on an electronically braked upright cycle ergometer (Ergoselect 100, Ergoline) with continuous 12-lead ECG monitoring. Blood pressure was recorded every two minutes and respired gas analysed in real time (Ultima CardiO2, Medical Graphics UK) for the assessment of gas exchange parameters. After three minutes of unloaded pedalling, a ramp protocol was applied. Participants were encouraged to continue until symptom-limited volitional fatigue, with a respiratory exchange ratio (RER) of >1.15 indicative of a good effort. Peak oxygen uptake (VO_2 peak_) was taken to be the mean O_2_ uptake in the final 20 seconds of the test, whilst O_2_ uptake at the anaerobic threshold (VO_2 AT_) was confirmed with the V-slope method and analysis of the ventilatory equivalents and end-tidal gas tension data [21].

#### Isometric muscle strength

Leg strength was measured using a hand-held dynamometer (MicroFET2 Torque/Force indicator, Hoggan Health Industries). Participants sat in an elevated chair with the lower leg positioned vertically and the knee flexed at 90 degrees. With the dynamometer held static against the lower shin, the participant exerted the maximal possible force by attempting to extend the knee against an equal and opposite resistance. Maximal contraction was performed three times on each leg with 30 seconds rest between each effort. Maximal quadriceps strength was reported as the mean of all six attempts.

#### Cardiac structure and function

With reference to the American Society of Echocardiography guidelines [22], resting transthoracic echocardiographic images were acquired and analysed (Vivid 7, Echo-pac version 7.0.0, GE Medical Systems) by a clinical sonographer blinded to group allocation. Left ventricular (LV) volumetric parameters, including LV ejection fraction (LVEF), were assessed in 2-D and calculated using the Simpson’s bi-plane method, indexed to body surface area. Left ventricular mass was calculated according to Lang et al (2015) [22]. In the apical four-chamber view, pulsed wave Doppler was employed to assess the ratio of early to late mitral inflow velocity (E/A) and E-wave deceleration time. Tissue Doppler imaging was used to quantify peak septal and lateral mitral annuli velocities. Subsequently, the ratio of peak early mitral inflow velocity to mean peak early annuli velocity (E/e’) was reported as a surrogate of LV filling pressure.

#### Arterial stiffness and endothelial function

Carotid-femoral pulse wave velocity (PWV) was derived with peripheral arterial applanation tonometry. Sequential recording of electrocardiogram-gated carotid and femoral waveforms was conducted with a high fidelity micromanometer (SPC-301, Miller Instruments) and processed via a mathematical transfer function (Sphygmacor, AtCor Medical Pty). The mean of three values was reported, and measurements conformed to pre-specified indicators of fidelity. For the assessment of endothelial function, established methodological and technical guidance for flow-mediated dilation (FMD) of the brachial artery was followed [23]. The endothelial response to hyperaemia was measured with duplex ultrasound imaging (Acuson P50, Siemens Medical, and Camberley, UK) of the non-fistula arm, prior to and following five minutes cuff inflation, distal to the imaging site.

#### Exercise interventions

Participants completing intra-dialytic cycling or LF-EMS were continually supervised by Clinical Exercise Physiologists. Both interventions were completed three times weekly for 10 weeks, for up to one hour. Exercise training was initiated after at least 30 mins of dialysis had elapsed and completed before the fourth hour. To ensure safety, heart rate and blood pressure were monitored throughout. Where sessions were missed, a two-week study extension was permitted to allow participants to accumulate sufficient exercise time.

#### Intra-dialytic cycling protocol

Semi-recumbent cycling (UBE-BD, Hudson Fitness) was performed whilst seated on a dialysis chair. Prior to commencing the 10-week programme, participants completed a two-week familiarization period, progressing to at least 30 mins of exercise. Thereafter, cycling was performed for up to one hour per session (minimum of 50 mins), initially at a workload (Watts) equivalent to that achieved at 40–60% VO_2_ reserve during CPET. Exercise intensity was regulated using HR and rating of perceived exertion (RPE). Participants were encouraged to exercise to an RPE of 12–14 with workload adjusted weekly by the supervisor to ensure a sufficient stimulus was achieved as exercise tolerance improved. Workload was controlled with pedal resistance and cadence to provide a personalised exercise prescription. A five-minute warm-up and cool down were performed at each session, with an extended cool down advised where a risk of post-exercise hypotension was identified.

#### Low-frequency electrical muscle stimulation protocol

Electrical muscle stimulation was delivered by four large (800cm^2^ total per leg), adhesive electrodes in a neoprene garment, applied bilaterally to the quadriceps and hamstrings. Details of the stimulation protocol have been previously described [24]. Briefly, to elicit a cardiovascular stimulus via rapid, rhythmical, sub-tetanic contractions, short bursts of four pulses were repeatedly delivered by a research stimulator (NT2010 Biomedical Research Ltd) at a frequency of 5 Hz. Current amplitude (strength of pulse) was adjustable from 5–140 mA with an inbuilt controller, accessible to both supervisor and participant. Two weeks of familiarization allowed participants to become accustomed to the sensation of LF-EMS and progress to at least 30 minutes of stimulation. The intervention was subsequently conducted for one hour at the maximum tolerable intensity [25], and participants (initially under supervision) were encouraged to increase the current amplitude to achieve a level of stimulation sufficient to evoke an increase in HR, BP, respiratory rate and body temperature. A five-minute warm-up and cool down at a lower frequency (4 Hz) were automatically applied by the stimulator. At higher stimulation intensities, excessive leg movement was attenuated by the participants pushing their feet against the foot rest of the dialysis chair.

#### Usual care (non-exercise control group)

Participants in the usual care group were advised to continue with their current level of daily physical activity, and their dialysis treatment, without intra-dialytic exercise. Commencement of additional supervised physical activity was discouraged.

### Statistical analyses

To assess and evaluate the feasibility of intra-dialytic cycling and LF-EMS, recruitment and retention rates, including 95% confidence intervals (95% CI) and statistical significance were examined. Baseline data were analysed to compare characteristics and clinical profile at trial entry, using means and confidence intervals for continuous variables and numbers (%) for categorical variables. Analyses of primary and secondary clinical outcomes were restricted to patients with baseline (week 0) and follow-up (week 10) measurements, comprising a "per protocol" analysis. Primary and Secondary clinical outcomes data were modelled using grand mean centred baseline measures as covariates. Mixed models using restricted maximum likelihood (REML) estimation and a variance components correlation structure were used to analyse outcomes, with baseline measures treated as random effects. Randomization group (the main factor of interest) plus age and sex were included in all mixed models as fixed effects. Final model based Least Squares Means (LS Means) for all three groups are presented for exercise capacity and clinical and cardiovascular outcomes, considered along with 95% CI. Differences between the groups modelled effects are presented with 95% CI and P-values. Analyses were performed with ‘R’. Statistical significance was indicated by *P*<0.05.

## Results

### Recruitment and participants

**Fig 1:**
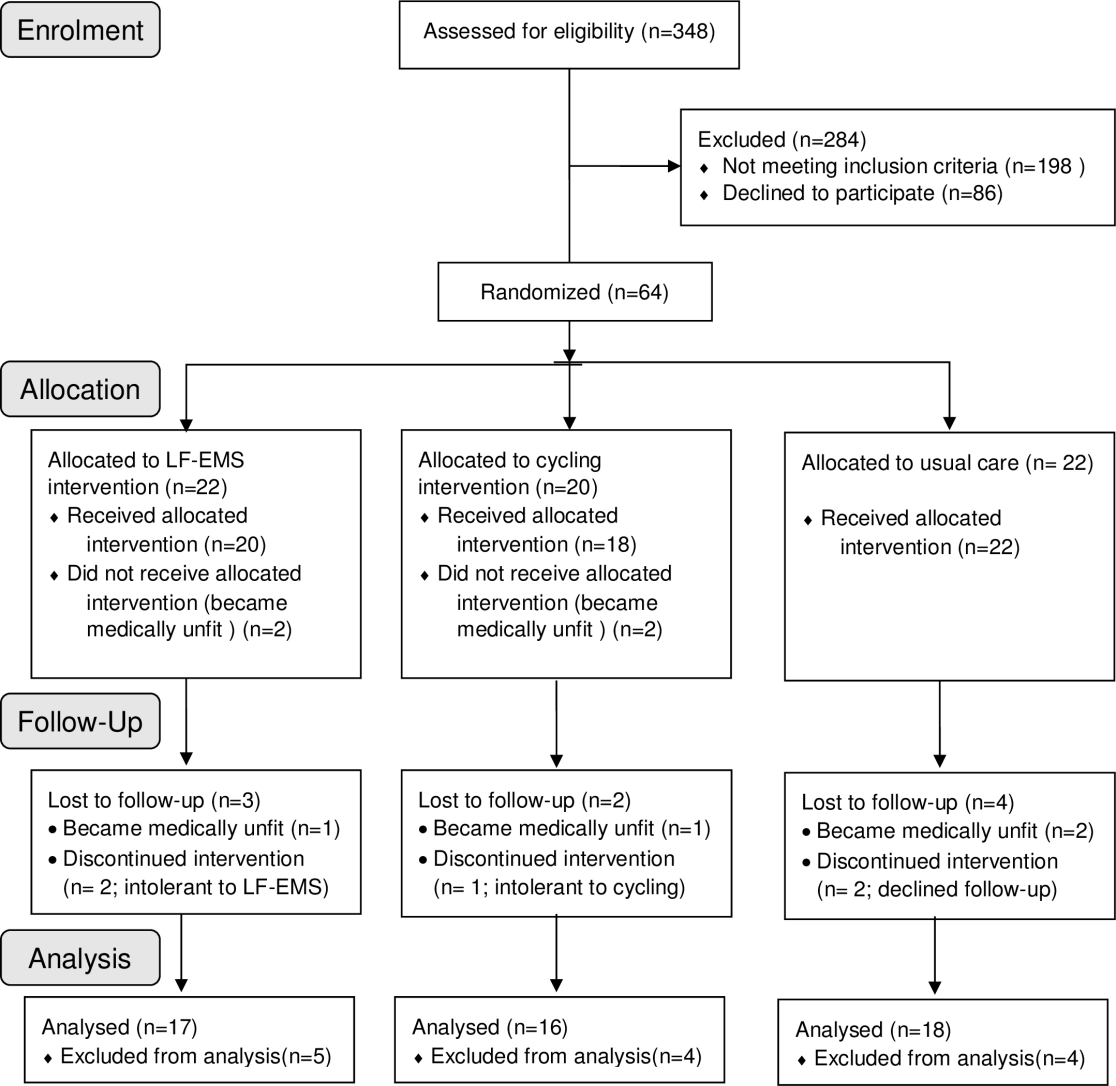
CONSORT (consolidated standards of reporting trials) diagram. LF-EMS, low frequency electrical muscle stimulation.

A total of 64 participants were randomized; cycle training (n = 22), LF-EMS (n = 20) and usual care (n = 22). A detailed breakdown of recruitment is provided in **Fig 1**. Demographics, clinical parameters and exercise capacity did not differ between groups at baseline **(Tables 1, 2 and 3)**. During the trial, there were no significant changes in medication.

**Table 1:** Baseline clinical characteristics.

	Control (n = 18)	Cycling (n = 16)	LF-EMS (n = 17)
Age (years)	54.3 [46.0; 62.5]	52.1 [44.2; 59.9]	51.5 [42.3; 60.6]
Sex, male	11 (61)	13 (81)	14 (82)
Weight (kg)	78.5 [68.3; 88.6]	85.1 [73.8; 96.5]	73.6 [67.3; 79.9]
BMI (kg/m^2^)	27.5 [24.6; 30.37]	29.2 [25.2; 33.2]	24.3[22.5; 26.1]
**Ethnicity, n (%)**			
Asian	6 (33)	4 (25)	2 (12)
Black	2 (11)	4 (25)	2 (12)
Caucasian	10 (56)	8 (50)	13 (76)
Hypertension, n (%)	12 (67)	12 (75)	13 (76)
Diabetes, n (%)	5 (28)	7 (44)	12 (71)
Smoking history, n (%)	7 (39)	7 (44)	12 (71)
CVD, n (%)	1 (6)	2 (13)	3 (18)
**ESRD aetiology n (%)**			
Glomerulonephritis	5 (28)	4 (25)	4 (23)
Diabetic Nephropathy	3 (16)	4 (25)	0 (0)
Vasculitis	4 (25)	0 (0)	2 (12)
Hypertensive Nephropathy	2 (11)	2 (13)	4 (23)
Pyelonephritis	1 (19)	1 (6)	4 (23)
Hereditary Nephropathy	0 (0)	1 (6)	2 (12)
Unknown	3 (16)	4 (25)	1 (6)
Dialysis vintage (months)	49.3 [29.6; 69.0]	48.1 [26.2; 70.0]	56.4 [36.1; 76.6]
**Medication**, n (%)			
Anti-hypertensive	13 (72)	13 (81)	13 (77)
Anti-diabetic	5 (28)	7 (44)	12 (71)
Anti-lipid	6 (33)	9 (56)	9 (53)
Iron treatment	2 (11)	5 (31)	4 (24)
Erythropoietin	11 (61)	9 (56)	15 (88)
Phosphate binder	10 (56)	14 (88)	15 (88)
Vitamin D	14 (78)	13 (81)	12 (71)
**Laboratory**		
Transferrin saturation (%)	25.4 [19.1; 31.6]	22.8 [18.8; 26.7]	24.9 [19.8; 29.9]
Hemoglobin (g/L)	114.7 [108.3; 121.1]	110.7 [104.3; 117.2]	119.9 [114.7; 125.1]
Creatinine (μmol/L)	707.8 [611.5; 804.1]	710.6 [614.9; 806.4]	715.5 [567.4; 863.5]
Corrected calcium (mmol/L)	2.2 [2.1; 2.2]	2.2 [2.1; 2.3]	2.3 [2.2; 2.4]
Phosphate (mmol/L)	1.3 [1.3; 1.7]	1.4 [1.3; 1.6]	1.5 [1.2; 1.7]
Albumin (g/L)	43.2 [41.2; 45.2]	46.0 [43.9; 48.1]	44.6 [42.7; 46.5]
hs-CRP (mg/L)	9.3 [4.6; 14.0]	5.3 [1.1; 9.4]	6.8 [3.2; 10.4]

Values are mean [CI] or frequency (%) for categorical variables. LF-EMS, low-frequency electrical muscle stimulation; BMI, body mass index; CVD, cardiovascular disease; ESRD, end stage renal disease; URR, urea reduction ratio.

**Table 2:** Exercise capacity at baseline and 10 weeks.

	Baseline			Week 10		
	Control	Cycling	LF-EMS	Control	Cycling	LF-EMS
HR rest (b.min^-1^)	81.11 [72.90; 89.32]	77.50 [69.30; 85.70]	81.06 [75.07; 87.05]	80.94 [74.66; 87.23]	74.00 [66.73; 81.27]	83.35 [75.59; 91.12]
HR peak (b.min^-1^)	121.28 [105.61; 136.94]	124.06 [112.84; 135.29]	120.38 [111.12; 129.64]	122.50 [109.02; 135.98]	126.88 [114.27; 139.48]	132.31 [119.96; 144.67]
VO_2 AT_ (ml.kg^-1^.min^-1^)	10.47 [8.99; 11.94]	11.30 [9.39; 13.22]	11.06 [9.62; 12.51]	10.17 [8.93; 11.40]	13.02 [11.12; 14.93]	12.47 [10.90; 14.04]
VO_2_ peak (ml.kg^-1^.min^-1^)	16.30 [13.63; 18.97]	18.25 [14.80; 21.70]	19.66 [16.29; 23.04]	15.93 [13.36; 18.50]	20.71 [17.07; 24.36]	20.97 [17.37; 24.57]
RER at VO_2 AT_	0.95 [0.91; 0.99]	0.97 [0.95; 0.99]	0.93 [0.90; 0.96]	0.94 [0.91; 0.96]	0.97 [0.95; 1.00]	0.96 [0.93; 0.98]
RER at VO_2_ peak	1.14 [1.11; 1.18]	1.26 [1.17; 1.34]	1.24 [1.17; 1.31]	1.12 [1.09; 1.15]	1.23 [1.16; 1.29]	1.26 [1.20; 1.32]
Max. load (Watts)	83.28 [70.29; 96.27]	100.50 [77.80; 123.20]	96.71 [83.58; 109.84]	79.22 [65.21; 93.24]	116.38 [95.01; 137.74]	108.71 [94.27; 123.14]
Leg Strength (Newtons)	401.03 [336.15; 465.90]	458.28 [352.44; 564.12]	420.15 [350.75; 489.55]	411.11 [341.74; 480.48]	530.34 [427.26; 633.43]	526.44 [448.13; 604.75]

Values are mean [CI]. LF-EMS, low-frequency electrical muscle stimulation; HR, heart rate; VO_2_, oxygen uptake; AT, anaerobic threshold;; RER, respiratory exchange rate

**Table 3:** Cardiac and vascular measures at baseline and 10 weeks.

	Baseline			Week 10		
	Control	Cycling	LF-EMS	Control	Cycling	LF-EMS
**Cardiac**						
LVMI (g/m^2^)	111.65 [91.86; 131.45]	139.20 [114.89; 163.52]	127.06 [87.00; 167.13]	112.18 [94.10; 130.27]	150.78 [128.20; 173.36]	123.69 [84.43; 162.95]
LVEDVI (ml/m^2^)	46.27 [40.88; 51.66]	52.37 [43.95; 60.79]	48.57 [41.22; 55.92]	46.28 [41.38; 51.17]	54.10 [45.49; 62.71]	46.88 [39.19; 54.57]
LVESVI (ml/m^2^)	22.03 [18.35; 25.71]	24.40 [18.58; 30.22]	20.31 [15.00; 25.61]	21.87 [18.63; 25.11]	25.17 [19.60; 30.75]	22.17 [17.23; 27.10]
LVEF (%)	52.91 [47.95; 57.86]	54.62 [48.97; 60.26]	58.75 [52.23; 65.27]	53.22 [49.68; 56.75]	54.22 [48.98; 59.45]	52.29 [44.70; 59.88]
E/A ratio	1.07 [0.79; 1.35]	1.11 [0.84; 1.39]	1.13 [0.77; 1.49]	1.00 [0.81; 1.19]	1.03 [0.86; 1.20]	1.14[0.78; 1.51]
Mean E/e’	10.89 [8.96; 12.81]	10.88 [8.05; 13.72]	9.43 [7.21; 11.64]	8.24 [5.87; 10.61]	11.93 [9.57; 14.30]	8.60 [7.09; 10.10]
LA diameter (cm)	3.89 [3.50; 4.27]	4.28 [3.92; 4.64]	3.95 [3.44; 4.47]	3.89 [3.52; 4.26]	4.29 [3.95; 4.64]	3.91 [3.43; 4.39]
**Vascular**						
SBP Rest (mm/Hg)	119.33 [106.82; 131.84]	130.06 [116.24; 143.88]	117.12 [102.71; 131.53]	123.17 [108.49; 137.84]	135.75 [122.28; 149.22]	126.71 [114.37; 139.04]
DBP Rest (mm/Hg)	69.06 [61.43; 76.68]	72.88 [62.45; 83.30]	67.41 [60.42; 74.40]	70.50 [62.30; 78.70]	72.13 [63.79; 80.46]	69.35 [59.50; 79.20]
PWV	8.47 [7.59; 9.35]	8.68 [7.31; 10.06]	7.69 [6.73; 8.65]	8.61 [7.82; 9.40]	8.14 [6.88; 9.41]	7.84 [6.87; 8.81]
FMD Delta (cm)	0.026 [0.018; 0.033]	0.023 [0.016; 0.030]	0.023 [0.014; 0.031] [0.014; 0.031]	0.031 [0.024; 0.037]	0.022 [0.016; 0.028]	0.027 [0.020; 0.035]
FMD Delta (%)	6.26 [4.29; 8.23]	5.61 [3.76; 7.45]	5.33 [3.42; 7.24]	7.55 [5.75; 9.35]	5.07 [3.48; 6.66]	6.53 [5.04; 8.01]

Values are mean [CI]. LF-EMS; low-frequency electrical muscle stimulation, LV, left ventricular; LVMI, LV mass index; LVEDVI, LV end diastolic volume index; LVESI, LV end systolic volume index; LVEF, LV ejection fraction; E/A ratio, ratio of peak early (E) to late (A) mitral inflow velocity; E/e’, ratio of peak early mitral inflow velocity to peak early diastolic mitral annulus tissue velocity; LA, left atrium; SBP, systolic blood pressure; DBP, diastolic blood pressure; PWV, pulse wave velocity; FMD, flow mediated dilatation

### Feasibility of LF-EMS and cycling

Three hundred and forty-eight patients were screened, of whom 150 were eligible (43.1%) and 64 recruited (42.7% of eligible patients) **(Fig 1)**. The primary exclusion/non-participation reason was the inability to complete CPET and cycle training due to comorbidity, musculo-skeletal limitation or low motivation. Of the 64 randomized participants, four were unable to commence exercise due to new medical problems. A further nine participants were excluded from the final analysis due to becoming medically unfit during the study (i.e. not meeting study inclusion criteria) (n = 4), declining follow-up (n = 2), or reporting intolerance to the intervention (n = 3). Therefore, the overall trial retention rate was 79.7%. Of those who dropped out due to intervention intolerance, one was unable to tolerate cycling (5.6%), and two LF-EMS (10.0%). The proportion of intolerance drop-outs did not differ between groups (*p* = 0.9). No patients in either group experienced post-exercise hypotension.

Adherence to exercise was excellent in both groups. In total, 91.0 ± 0.1% of sessions were completed in the LF-EMS group and 93.0 ± 0.1% in the cycling group. Mean exercise time and intensity achieved by week 10 was 56.3 ± 6.7 mins and 63.8 ± 20.7 watts (equivalent to 63.8% VO_2peak_ from CPET) for cycling and 60.0 ± 0.1 mins and 119.7 ± 13.0 mA for LF-EMS.

### Clinical assessment and anthropometry

Ten weeks of cycling or LF-EMS did not alter any clinical parameters **(Table 1)**.

### Exercise capacity

**Fig 2:**
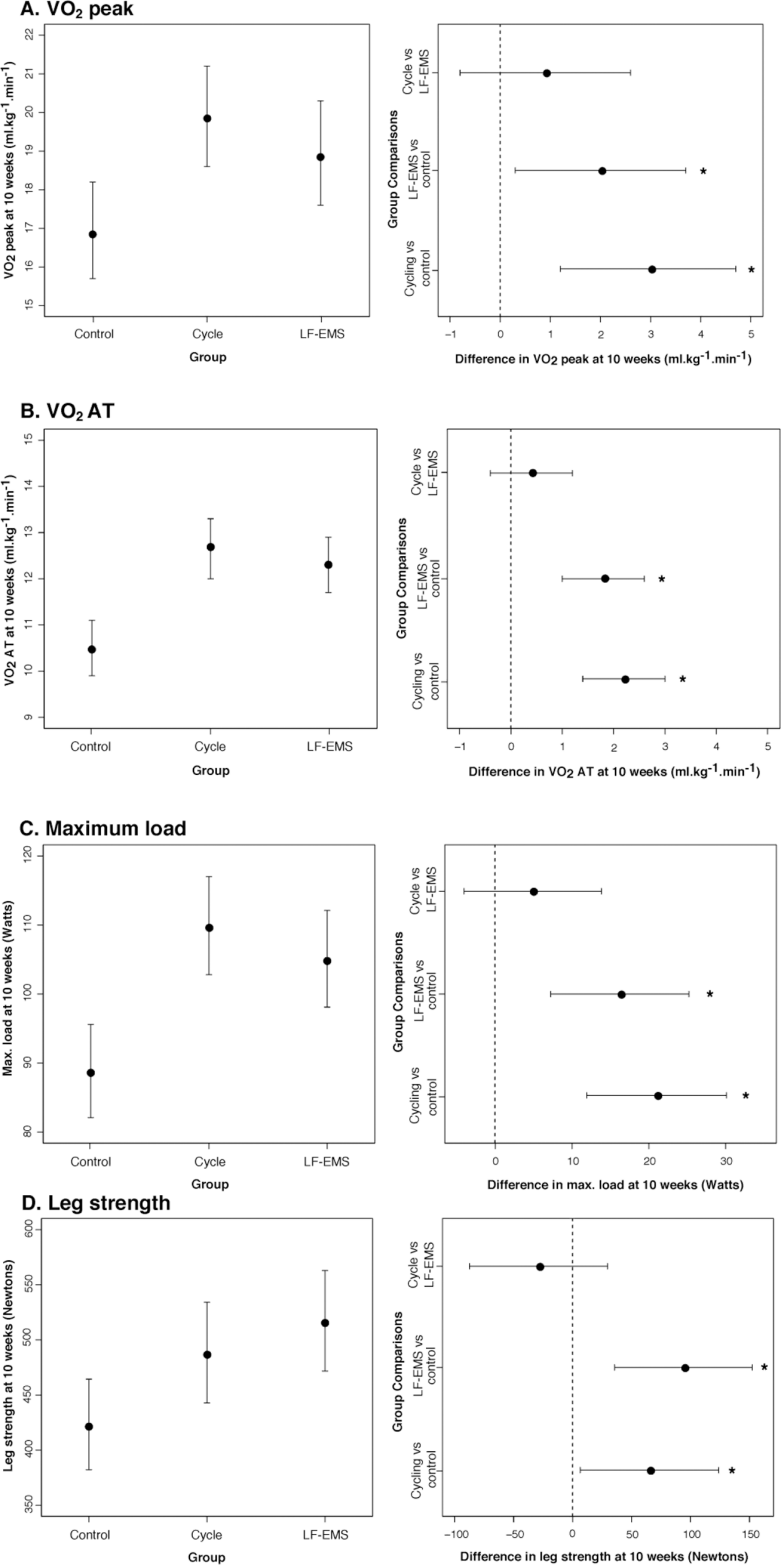
Least squares means at 10 weeks adjusted for baseline value, age and sex (left panels), and difference between groups in least squares means at 10 weeks (right panels) for (A) peak oxygen uptake (VO_2_ peak); (B) oxygen uptake at anaerobic threshold (VO_2 AT_); (C) maximum load (max. load); and (D) leg strength. Right hand panels show the superiority of cycling and LF-EMS over control for all variables (A-E) and no difference between cycling and LF-EMS. LF-EMS, low-frequency electrical muscle stimulation; *P<0.05.

Both interventions significantly improved cardio-respiratory reserve and muscular strength **(Table 4)**. At 10 weeks, comparing modelled mean changes, adjusted for baseline value, age and sex, indicated the superiority of LF-EMS and cycling over control for VO_2 peak,_ VO_2 AT_, maximum workload and isometric leg strength **(Table 4 and Fig 2)**. LF-EMS and cycling improved VO_2 peak_ (+2.0 [95% CI, 0.3 to 3.7] ml.kg^-1^.min^-1^, *P* = 0.02, and +3.0 [95% CI, 1.2 to 4.7] ml.kg^-1^.min^-1^, *P* = 0.001 respectively), VO_2 AT_ (+1.8 [95% CI, 1.0 to 2.6] ml.kg^-1^.min^-1^, *P* = 0.02, and +2.2 [95% CI, 1.4 to 3.0] ml.kg^-1^.min^-1^, *P* = 0.001 respectively), maximum workload (+16.2 [95% CI, 7.2 to 25.2] ml.kg^-1^.min^-1^, *P* = 0.02, and +21.0 [95% CI, 11.9 to 30.1] ml.kg^-1^.min^-1^, *P* = 0.001 respectively), and leg strength (+94 [95% CI, 35.6 to 152.3] N, *P* = 0.002, and +65.1 [95% CI, 6.4 to 123.8] N, *P* = 0.002 respectively) **(Fig 2)**. Neither intervention demonstrated significantly greater benefit over the other for all measures of exercise capacity (*P*<0.05), as shown in the LF-EMS vs. cycling comparisons **(Fig 2)**.

**Table 4:** Exercise capacity, cardiac and vascular measures at 10 weeks adjusted for baseline values, age and sex.

	Least squares estimates at week 10						
	Control	Cycling	LF-EMS	P*	*P*^*‡*^ (Cycling vs. LF-EMS)	*P*^*‡*^ (Cycling vs. Control)	*P*^*‡*^ (LF-EMS vs. Control)
**Exercise Capacity**							
HR rest (b.min^-1^)	80.90 [76.49; 85.32]	77.09 [72.00; 82.18]	83.85 [78.90; 88.81]	0.1	0.04	0.2	0.4
HR peak (b.min^-1^)	123.44 [116.69; 130.20]	126.65 [118.84; 134.45]	135.44 [127.46; 143.42]	0.06	0.09	0.5	0.02
VO_2 AT_ (ml.kg^-1^.min^-1^)	10.50 [9.94; 11.05]	12.66 [12.03; 13.29]	12.30 [11.67; 12.92]	<0.001	0.4	<0.001	<0.001
VO_2_ peak (ml.kg^-1^.min^-1^)	16.93 [15.69; 18.17]	19.88 [18.55; 21.21]	18.94 [17.62; 20.26]	0.004	0.3	0.001	0.02
RER at VO_2 AT_	0.93 [0.91; 0.96]	0.96 [0.94; 0.99]	0.95 [0.92; 0.97]	0.2	0.4	0.09	0.4
Max. load (Watts)	88.87 [82.10; 95.64]	109.89 [102.82; 116.97]	105.08 [98.09; 112.07]	<0.001	0.3	<0.001	0.001
Leg Strength (Newtons)	423.32 [382.28; 464.37]	488.45 [442.82; 534.09]	517.28 [471.65; 562.92]	0.007	0.3	0.03	0.002
**Cardiac**							
LVMI (g/m^2^)	111.60 [88.09; 135.11]	138.99 [110.98; 167.00]	114.95 [87.00; 142.90]	0.2	0.2	0.1	0.8
LVEDVI (ml/m^2^)	47.84 [43.33; 52.35]	50.37 [45.61; 55.12]	44.33 [38.31; 50.34]	0.2	0.09	0.4	0.3
LVESVI (ml/m^2^)	21.97 [19.43; 24.52]	23.14 [20.32; 25.95]	21.12 [17.45; 24.78]	0.6	0.3	0.5	0.7
LVEF (%)	53.59 [49.57; 57.61]	54.10 [49.78; 58.41]	50.15 [44.37; 55.94]	0.5	0.2	0.9	0.3
E/A ratio	1.04 [0.82; 1.26]	1.05 [0.81; 1.29]	1.16 [0.91; 1.41]	0.7	0.5	0.9	0.5
Mean E/e’	8.52 [6.31;10.72]	12.06 [9.95;14.17]	8.90 [6.65;11.16]	0.04	0.05	0.03	0.8
LA diameter (cm)	3.93 [3.61; 4.24]	4.06 [3.69; 4.44]	3.82 [3.45; 4.18]	0.6	0.3	0.6	0.6
**Vascular**							
SBP Rest (mm/Hg)	126.26 [115.51; 137.00]	136.88 [124.54; 149.23]	130.19 [117.93; 142.45]	0.4	0.4	0.2	0.6
DBP Rest (mm/Hg)	71.20 [64.41; 78.00]	70.48 [62.73; 78.23]	69.89 [62.18; 77.59]	0.9	0.9	0.8	0.8
PWV	8.45 [7.76; 9.14]	7.94 [7.19; 8.70]	8.17 [7.40; 8.94]	0.6	0.6	0.3	0.6
FMD Delta (cm)	0.03 [0.02; 0.04]	0.02 [0.02; 0.03]	0.03 [0.02; 0.03]	0.08	0.1	0.03	0.6
FMD Delta (%)	7.54 [6.26; 8.82]	5.29 [3.90; 6.69]	6.96[5.51; 8.40]	0.05	0.08	0.02	0.5

Values are mean [CI] at 10 weeks adjusted for baseline value, age, and sex. LF-EMS, low-frequency electrical muscle stimulation; HR, heart rate; VO_2_, oxygen uptake; AT, anaerobic threshold; RER, respiratory exchange ratio; LV, left ventricular; LVMI, LV mass index; LVEDVI, LV end diastolic volume index; LVESI, LV end systolic volume index; LVEF, LV ejection fraction; E/A ratio, ratio of peak early (E) to late (A) mitral inflow velocity; E/e’, ratio of peak early mitral inflow velocity to peak early diastolic mitral annulus tissue velocity; LA, left atrium; SBP, systolic blood pressure; DBP, diastolic blood pressure; PWV, pulse wave velocity; FMD, flow mediated dilatation. P*, between all 3 groups; *P*^***‡***^, between two specified groups.

### Cardiovascular structure and function

Arterial structure and function were unaffected by cycling or LF-EMS. Neither arterial stiffness, measured by carotid-femoral PWV, or endothelial function, measured by FMD, changed significantly over the 10-week study period **(Table 4)**. Echocardiographic parameters remained stable in all three groups. However, E/e’ (surrogate of LV filling pressure) was significantly improved by cycling compared to both LF-EMS and control **(Table 4)**.

## Discussion

Reduced exercise capacity is a prominent feature in CKF. The ability to attenuate functional decline is of great importance to combat deteriorating quality of life and morbidity. Rehabilitative strategies are poorly defined despite promising results with intra-dialytic exercise. A particular challenge is the provision of exercise therapy for those who are unable to perform conventional dynamic training. Here we have compared the feasibility of two intra-dialytic exercise interventions; LF-EMS and cycling. Our data show that both interventions were feasible, whilst equally improving cardio-pulmonary functional capacity and muscle strength.

As the primary outcome measure for this pilot study, the feasibility of LF-EMS and cycling during dialysis was assessed with recruitment rates, intervention tolerability and exercise session adherence. To be eligible, potential participants had to be willing and able to undertake maximal exercise testing, intra-dialytic cycling and LF-EMS. During recruitment, LF-EMS was rarely contra-indicated or cited as a barrier to enrolment. Conversely, dynamic exercise testing and training was the main reason for study ineligibility and unwillingness to participate. Future LF-EMS trials are likely to recruit larger numbers if intra-dialytic cycling is not included as a treatment arm, or CPET as an outcome measure. In relation to intervention tolerability, cycling and LF-EMS were comparable. Only one dropout in the cycling group (5.6%), and two in the LF-EMS group (10.0%) were due to intolerance. Likewise, exercise session compliance was very high; above 90% in both intervention groups. With minimal drop-out due to intolerance, high exercise session adherence and no reports of adverse events, both exercise protocols can be regarded as feasible, and LF-EMS appears to be a suitable treatment arm for a definitive clinical trial.

The potential for dynamic intra-dialytic exercise (cycling and/or resistance training) to impact favorably on functional measures is relatively well known in CKF. Specifically, VO_2 peak_, six-minute walk distance and self-reported physical functioning have been shown to improve with intra-dialytic exercise [11, 12, 26]. Consistent with our data, gains of 10–15% in VO_2 peak_ can been expected, however, training of at least three months, and preferably six, are optimal [11, 12, 26]. Given the short 10-week duration of our study, the observed VO_2 peak_ improvement of 13% is impressive. This is likely the result of using CPET data to objectively prescribe exercise intensity combined with weekly titration of exercise dose and close supervision during training sessions.

The use of low-frequency electrical muscle stimulation during hemodialysis is a novel approach to cardiovascular exercise training. To elicit a continuous cardiovascular response, we administered a unique low-frequency (4–5 Hz) protocol causing rhythmical sub-tetanic contraction of the quadriceps and hamstrings. This was preferred to more commonly adopted high-frequency protocols (30-70Hz), in which intermittent tetanic contraction is aimed specifically at increasing muscular strength. Rapid muscular fatigue is common with this approach and prolonged training unsustainable [27]. Whilst gains in muscle strength are undoubtedly a desirable outcome in CKF, cardiovascular exercise seems preferable to address not only muscular strength but also cardiovascular morbidity and mortality. An acute cardio-respiratory response to LF-EMS was observed in all study participants. Moreover, the documented improvement in VO_2 peak_ at 10 weeks indicates cardiovascular adaptation, with LF-EMS proving to be as effective as cycling. With a large proportion of patients unable or unwilling (>50% locally) to engage in dynamic intra-dialytic exercise, these data provide compelling evidence of an alternative exercise modality that is feasible and potentially effective.

The improved maximal exercise capacity (VO_2 peak_) with cycling and LF-EMS in our CKF cohort has the potential to improve patient survival and reduce morbidity [28]. Not only could renal transplant be made safer, but patients who were originally deemed unfit for surgery could improve their exercise capacity and be considered for transplantation. [28, 29]. Our group recently showed that reduced exercise capacity in patients with CKF was associated with premature death, and that improved exercise capacity after a renal transplant resulted in improved survival [28]. However, whilst VO_2 peak_ is a powerful indicator of survival, it is difficult to measure accurately in debilitated, comorbid patients [30]. Our data show that LF-EMS and cycling also improved anaerobic threshold (VO_2 AT_) by 15%. As a measure of aerobic capacity, VO_2 AT_ is considered to be more objective than VO_2 peak_, as it is not influenced by patient effort and motivation. As an indicator of greater skeletal muscle oxidative capacity, VO_2 AT_ also has important clinical implications [30], both in terms of predicting survival in CKF [28] and assessing the mortality risk of major surgery [28–33]. For those unable to undertake cycle training, the potential for LF-EMS to increase VO_2 AT_, thus providing a route to renal transplantation, is encouraging. These results should be considered hypothesis generating for future interventional studies.

In addition to cardiopulmonary adaptation, we observed significant gains in quadriceps strength in both treatment groups; 17% and 24% with cycling and LF-EMS, respectively. Intra-dialytic exercise has previously been associated with increased muscle strength [12, 34]. The insidious decline in lean muscle mass and function in CKF is a complex clinical entity mediated, in part, by metabolic acidosis, protein energy wasting and circulating inflammatory cytokines [35]. Markedly impaired physical functioning results, and a powerful association exists with mortality [36, 37]. Novel, therapeutic strategies to combat such deleterious outcomes are warranted. The 24% improvement in quadriceps strength witnessed with LF-EMS is likely to have a profound effect on everyday physical functioning and potentially reduce the risk of falls in the elderly. Our data does not elucidate the mechanisms by which such a large effect was achieved. Whilst the sub-tetanic muscle activation protocol was designed to improve aerobic capacity, it also appears to have had a localised effect on anaerobic performance. Aerobic exercise commonly facilitates moderate improvements in muscular strength. The exaggerated response we observed is likely explained by oxygen uptake kinetics in LF-EMS. Previous work has shown the predominance of anaerobic metabolism in the first 15 mins of stimulation, with a greater reliance on aerobic metabolism thereafter [15, 38, 39]. As such, the intervention may have included a muscular strength training stimulus in addition to the aerobic component. With such a magnitude of change in the context of deranged muscle in CKF, our data promote the adoption of LF-EMS to maximise strength gains from exercise training in CKF.

In CKF, arterial stiffness and endothelial dysfunction are independent predictors of cardiovascular mortality and contribute to exercise intolerance [40–42]. Thickening of the intimal and medial arterial layers due to increased collagen content, hyperplasia and hypertrophy of the vascular smooth muscle cells, combines with concentric calcification to increase arterial stiffness [43]. Furthermore, systemic inflammation and oxidative stress reduce the bioavailability of nitric oxide [44], thus impairing endothelial derived vasodilation [45]. Exercise induced vascular adaptation is widely reported in healthy and clinical populations, the extent of which correlates with improved exercise capacity [46]. In the present study, we did not observe any improvement in vascular function, although this may be unsurprising, as the study was not powered to detect such a change. Pulse wave velocity and FMD, both of which were severely reduced at baseline, were unaffected by cycling or LF-EMS. Previous data in hemodialysis patients is inconclusive; arterial stiffness is either unchanged or reduced with training [14, 47–49], whilst FMD has not been investigated. In CKD stages 3–4, the absence of a training effect on PWV, FMD and cellular markers of endothelial function has also been reported [50]. Although exploratory, our data may corroborate these findings suggesting that exercise induced vascular adaptation is not responsible for improved VO_2 peak_ in CKF. The implications of these preliminary findings are unclear but a beneficial training effect appears to exist, independent of altered vasculature.

Regarding cardiac structure and function, little overall change was observed. In comparison to both control and LF-EMS, however, cycling did significantly improve LV diastolic filling pressure, as quantified by E/e’. As a single measure in isolation, it is difficult to confirm meaningful cardiac adaptation with intra-dialytic cycling, but this area is currently under investigation elsewhere [51]. Data from CKF and heart failure populations confirm E/e’ to be a key determinant of exercise capacity which can be improved with exercise training [3, 52]. Marked cardiac dysfunction is evident in CKF and exercise therapy should aim to address this.

### Considerations for a future trial

This randomised pilot study, with small patient groups, produced promising results, particularly in relation to the LF-EMS treatment intervention. Whilst we observed improvements in several clinical parameters, we must acknowledge the exploratory nature of these findings, and the likelihood of type 1 error inflation associated with multiple hypothesis testing. Future multi-centre clinical trials should focus exclusively on LF-EMS, and evaluate exercise capacity, quality of life, morbidity, and mortality. With VO_2 AT_ as a candidate primary outcome, a definitive trial comparing LF-EMS with usual care, would require 83 participants per group [53]. With 90% power and alpha set at 5%, this sample size is based on detecting a difference of 1.75 ml.kg^-1^.min^-1^, with an SD of 3.09 ml.kg^-1^.min^-1^ (as per current trial) and a drop-out rate of 20%. For a multi-centre RCT, stratification by site might be desirable, potentially leading to a larger sample size. Data and experience from the present and subsequently published trials will inform design and implementation.

A number of limitations warrant discussion. Most importantly, data from this trial is preliminary and must be interpreted with caution. Also, recruitment was restricted to patients who could complete cycle training and most participants were male (80%). Therefore, the findings cannot be generalized to the wider CKF population. The intervention period was short (10 weeks), and it remains to be confirmed if compliance and adherence can be maintained in the longer term.

## Conclusions

In conclusion, our study highlights the feasibility and potential efficacy of intra-dialytic LF-EMS and cycle training. The improved VO_2 peak_, VO_2 AT_ and muscular strength we observed are important clinical and functional outcomes for patients on hemodialysis. For the many instances in which conventional dynamic exercise is prevented by comorbidity and fatigue, we propose the use of LF-EMS as a suitable alternative in future exercise trials for CKF patients. This exercise modality can be easily administered on dialysis units.

## Supporting information

S1 File Dataset 1. Full trial dataset.
(XLSX)

S2 File Study protocol. (PDF)

S3 File CONSORT checklist. (DOC)
