# Supplementary material for: Feasibility and effects of intra-dialytic low-frequency electrical muscle stimulation and cycle training: A pilot randomized controlled trial
Source: PLoS One. 2018 Jul 11;13(7):e0200354. doi: 10.1371/journal.pone.0200354 (PMC6040736; doi:10.1371/journal.pone.0200354)
Supplement: S2 File — (PDF) [file pone.0200354.s002.pdf]

PROTOCOL

Kidney disease mediated cardiac and skeletal muscle impairment: effects of dynamic exercise training and low frequency electrical muscle stimulation during haemodialysis; a pilot study.

Dr Gordon McGregor<br>Clinical Exercise Physiologist<br>Research Fellow<br>Renal Research Office<br>University Hospital<br>Clifford Bridge Rd<br>Coventry CV2 2DX<br>Tel: 07772 462 255<br>E-mail: gordon.mcgregor@uhcw.nhs.uk	Dr Prithwish Banerjee<br>Consultant Cardiologist<br>UHCW NHS Trust<br>University Hospital<br>Clifford Bridge Rd<br>Coventry CV2 2DX<br>Tel: 02476 965 670<br>E-mail: prithwish.banerjee@uhcw.nhs.uk
Dr Daniel Zehnder<br>Consultant Nephrologist & Associate Professor<br>University of Warwick<br>Warwick Medical School<br>Clinical Sciences Research Institute<br>Coventry CV2 2DX<br>Tel: 02476965689<br>E-mail: d.zehnder@warwick.ac.uk	Dr Nithya Krishnan<br>Consultant Nephrologist<br>UHCW NHS Trust<br>University Hospital<br>Clifford Bridge Rd<br>Coventry CV2 2DX<br>Tel:<br>E-mail: nithya.krishnan@uhcw.nhs.uk
Dr Stephen Ting<br>Renal Registrar<br>UHCW NHS Trust<br>University Hospital<br>Clifford Bridge Rd<br>Coventry CV2 2DX<br>Tel:<br>E-mail: stephen_ting@yahoo.com	Stuart Ennis<br>Clinical Exercise Physiologist<br>Cardiac Rehabilitation<br>Centre for Exercise & Health<br>Watch Close<br>Coventry CV1 3LY<br>Tel: 02476 234 570<br>E-mail: stuart.ennis@uhcw.nhs.uk

Background/rationale

The burden of cardiovascular disease in chronic kidney disease

Chronic kidney disease (CKD) affects 8.5% of the adult UK population with in excess of 40,000 patients requiring renal replacement therapy for end stage renal disease (ESRD)[1]. As both a cause and effect of CKD, coexistence of cardiovascular disease (CVD) is as high as 50%[2]. Hypertension, often in combination with diabetes, is the primary cardiovascular (CV) aetiology[3] but CV risk is increased even in the absence of traditional CVD risk factors[4, 5]. Cardiovascular complications represent the main factor limiting the overall survival of CKD populations, with 40% of deaths attributed to CV causes[6, 7]. Among these, chronic heart failure (CHF) contributes substantially to the high rates of premature death in this population. Recent analyses of dialysis patients highlighted CHF not only as a major risk factor for hospitalisation, but also as associated with 83% mortality at 3 years after hospitalisation[8]. Despite this, beneficial CVD treatments seem to be underused in patients with CKD, as are rehabilitation therapies such as exercise training[9]. This is likely due to a lack of data, as the majority of drug treatment trials which have shown reduced CV risk, have excluded patients with CKD[10]. Furthermore, uncertainty surrounds which of the many established or novel CV risk factors should be prioritised in this population.

The benefits of exercise training

The effects of physical activity and exercise in the general population are well documented. From the perspective of primary prevention of disease, participation in regular physical activity i.e 150 mins of moderate intensity physical activity per week, unequivocally reduces morbidity and mortality associated with CVD and cancers. Exercise training is also an effective therapeutic intervention in established disease[11-14]. As an integral component of rehabilitation in cardiac disease, appropriately prescribed exercise training has been shown to improve functional capacity[15-18] and reduce CV and all cause mortality by 25-30%[19]. A multifactorial mode of action is likely responsible, but the precise physiological mechanisms, and their relative contribution, remain to be confirmed. In addition to attenuated autonomic and neurohormonal activation, improvements in ventilatory efficiency, skeletal muscle metabolism and vascular endothelial function clearly contribute to improved functional capacity in this patient group[20]. Furthermore, in combination with the direct myocardial effects demonstrated in animal models (i.e. reduced hypertrophy, fibrosis and apoptosis through interference with maladaptive signalling pathways)[21-24], exercise may favourably impact upon pathological LV remodelling by reducing disease specific systemic dysfunction [25]. In post-MI and CHF patients, meta-analyses have reported decreased LV end systolic and diastolic volumes and increased LV ejection fraction (LVEF) further to moderate intensity (40-60% VO_2 reserve) exercise training interventions[25, 26]. A key feature of studies in these patient groups, however, is the exclusion of patients with CKD.

Cardiac remodelling in chronic kidney disease

In CKD, a severely reduced exercise capacity (commonly <60% of normal) and poor quality of life (QOL)[12] is precipitated by structural and functional abnormalities of the CV system and skeletal muscle, leading to a vicious circle of chronic fatigue and inactivity[12]. The pattern of CVD in ESRD patients differs from general patient populations with disproportionate CHF prevalence in the absence of significant coronary artery disease (CAD)[27]. Left ventricular hypertrophy (LVH), LV dilatation, myocardial fibrosis, diffuse arteriosclerosis, calcific atherosclerosis, calcific valves, diastolic and systolic dysfunction are common CV sequelae [27-29]. Left ventricular maladaptation is instigated by chronic hypertension[30], for which sodium retention and the activation of the renin-angiotensin system (RAS) and the sympathetic system are to blame[31]. In combination with renal anaemia and increased vascular stiffness, the development of concentric LVH leads to a significantly worsened prognosis[32] due to progressive CHF. Among patients with ESRD, 70% have LVH, 15% have systolic dysfunction and 40%

have CHF[30]. In addition to fibrotic LVH and CHF, abnormal electrolyte concentrations, arrhythmia and ischemic coronary artery disease lead to an increase in sudden cardiac death[33], although myocardial infarction accounts for relatively few events[34]. Further pathological mechanisms of increased CVD risk in CKD are extensive, including dyslipidaemia, systemic inflammation, decreased bioavailability of nitric oxide, increased RAS and sympathetic activity, impaired endothelial function, vitamin D deficiency, hyperparathyroidism and altered mineral metabolism[9, 28, 35]. Collectively, the complex interaction between the kidneys and the heart is known as the cardio-renal syndrome[36] with the destructive effects of CHF being the predominant feature.

Arterial remodelling in chronic kidney disease
The impact of arteriosclerosis is significant in CKD. Thickening of the medial arterial layer, due to increased collagen content, hyperplasia and hypertrophy of the vascular smooth muscle cells, combines with concentric calcification to increase arterial stiffness[37]. A strong association between arterial stiffness and mortality has been reported in ESRD[38]. Loss of arterial distensibility leads to increased risk of CHF, arrhythmia and stroke from microvascular damage caused by the higher systolic pressures required to maintain arterial hemodynamics[39]. Simultaneously, an early return of the reflected forward pressure wave to the central circulation causes a decreased diastolic pressure, thereby compromising myocardial perfusion via the coronary arteries[40]. Ultimately, the higher LV afterload resulting from aortic stiffness leads to increased LV mass[41], LV stiffness[42] and LV filling pressures[43], increasing myocardial O_2 consumption and energy expenditure. Cardiovascular reserve is reduced and hemodynamic stability compromised, exposing the myocardium to further LV structural maladaptation and functional impairment[44].

Endothelial dysfunction also occurs early in the arteriosclerotic process and is integral to the progression of arterial stiffness[45]. As a measure of endothelial function, flow-mediated vasodilation of the brachial artery is inversely related to arterial stiffness in hypertensive patients[46]. Impaired endothelial function has been demonstrated in the large and small arteries of CKD patients[46, 47], relating strongly to kidney and CVD outcomes[48, 49]. Chronic kidney disease related low grade inflammation, oxidative stress and hormonal factors appear to reduce the bioavailability of nitric oxide (NO)[45], a key protagonist of endothelial derived vasodilation. Arterial remodelling as a whole, involving increased stiffness and impaired flow-mediated vasodilation, is a key cardiovascular feature of CKD. In combination with cardiac maladaptation and skeletal muscle abnormalities this contributes to the significantly impaired exercise capacity observed in the CKD population.

Skeletal muscle dysfunction in chronic kidney disease
A further piece of the pathophysiological jigsaw dictating morbidity and mortality in CKD is skeletal muscle wasting[50]. The key driver of this process is an inflammatory cytokine and inactivity mediated imbalance in protein homeostasis, resulting in a catabolic destruction of structural and functional proteins with debilitating muscle wasting[51]. As kidney function declines and haemodialysis is initiated, poor nutritional status is mediated by anorexia, increased resting energy expenditure and a disproportionate increase in protein catabolism as a result of activation of the inflammatory cascade and proteolytic pathways[52]. Hypercatabolic protein energy wasting is a complex biomolecular process and the understanding of the phenomenon continues to evolve. Medical and pharmacological therapies to combat this state remain ineffective and it is clear that exercise training may be the most promising therapy[51].

Exercise training in chronic kidney disease
When conducted between dialysis sessions (inter-dialytic), dynamic CV exercise and strength training can improve catabolic destruction of proteins, muscular fitness, resting systolic and diastolic blood pressure

and QOL[12]. The most effective programmes appear to be supervised and longer than 4 months in duration, adopting a mixed CV and strength training approach at intensities >60% VO_2 reserve[12]. However, despite the high incidence of CHF in CKD, only three studies have examined the effect of exercise training on the heart[53-55], all of which reported no change in standard structural measures. Compliance and adherence to an inter-dialytic exercise regimen can be challenging for chronically fatigued patients[12]. Limited recent work has confirmed that exercise performed during dialysis (intra-dialytic) is safe and indicates favourable outcomes in functional capacity, muscular strength, physical activity participation, arterial compliance and biochemical markers (serum phosphate and potassium)[56-60]. Intradialytic exercise may therefore offer an effective alternative as appointment attendance is essential, supervision is readily available and time on dialysis is used effectively. To date, benefits gained from this approach have not been maximised as trials have been characterised by poorly designed and controlled exercise interventions with insufficient prior assessment of exercise capacity and inaccurate regulation of exercise intensity.

Low frequency electrical muscle stimulation (LF-EMS)

With chronic and progressive moypathic changes in cardiac and skeletal muscle, it is common for dynamic exercise to be unachievable in patients with ESRD[7]. In these patients, alternative exercise stimuli should be considered. Low frequency electrical muscle stimulation (LF-EMS) of the leg muscles is well tolerated and has been shown to produce similar benefits to aerobic training, increasing exercise capacity, reducing dyspnoea and improving QOL in chronic heart failure [61-63], chronic obstructive pulmonary disease[64] and other conditions. Neuromuscular electrical stimulation of this origin elicits rhythmical, subtetanic, isometric contractions of the quadriceps and hamstrings, mimicking the pattern of muscle activation associated with shivering[65]. Energy expenditure is increased leading to improvements in exercise capacity[66-68]. Intra-dialytic LF-EMS may offer a suitable alternative to dynamic intra-dialytic exercise. Indeed, comparable benefit has been demonstrated in the only previous study examining LF-EMS during dialysis[69]. Muscular strength, six minute walk distance, QOL and urea clearance were shown to be equally improved in patients completing 20 weeks of intra-dialytic dynamic exercise or LF-EMS. Importantly, LF-EMS was well tolerated and there were no adverse events throughout the duration of the study.

Summary

In a low functioning, inactive patient group, at increased risk of arterial and ventricular remodelling and skeletal muscle and other protein wasting, the development of safe and effective exercise strategies is essential to promote functional independence, improve QOL and potentially reduce mortality. An understanding of the CV and skeletal muscle adaptation to these interventions is required to quantify their impact and to inform effective exercise intervention design in CKD patients on dialysis. Through the use of validated techniques (echocardiography, cardiopulmonary exercise testing, leg dynamometry, physical activity monitoring, arterial applanation tonometry, flow mediated vascular dilatation, biochemistry, QOL questionnaire), comprehensive evaluation of the effects of intra-dialytic exercise training will likely provide insight into the most appropriate future strategies for the management of patients with ESRD, maximising the impact of these therapies in the context of the limited availability of effective alternative treatments.

Hypotheses:

1. Intra-dialytic exercise training is well tolerated and improves well-being in stable, chronic dialysis patients.
2. Intra-dialytic exercise training significantly improves cardiovascular and skeletal muscle function with reversal of the catabolic process.
3. The effect of intra-dialytic LF-EMS exercise on cardiovascular and skeletal muscle function and its tolerability is comparable to dynamic exercise training effects.

Aims are:

1. To assess the tolerability of intra-dialytic dynamic exercise and LF-EMS interventions compared to conventional treatment without exercise intervention in patients on regular dialysis for end stage renal failure.
2. To assess the improvement of quality of life with intra-dialytic dynamic exercise and LF-EMS interventions compared to conventional treatment without exercise intervention in patients on regular dialysis for end stage renal failure.
3. To evaluate the effects of intra-dialytic dynamic exercise training compared to conventional treatment without exercise intervention on measures of functional cardiovascular and skeletal muscle function.
4. To evaluate the effects of LF-EMS intra-dialytic exercise training compared to conventional treatment without exercise intervention on measures of functional cardiovascular and skeletal muscle function.
5. To qualitatively compare the effects of LF-EMS with dynamic intra-dialytic exercise training on measures of functional cardiovascular and skeletal muscle function.

Participants:

Patients on chronic and stable haemodialysis, under the renal care of Nephrologists at the University Hospital Coventry and Warwickshire NHS Trust will be invited to take part and will be randomised after informed consent is received.

Haemodialysis sites: Patients from five different sites under the care of the Nephrologists at the UHCW will be approached. At these sites, 450 patients are receiving regular dialysis.

1. University Hospital, Coventry, UHCW NHS Trust; Dialysis Unit
2. Rugby St. Cross Hospital, UHCW NHS Trust; Ash Dialysis Unit
3. The Whitnash Satellite Renal Unit; UHCW NHS Trust
4. Stratford Hospital, Stratford upon Avon, UHCW NHS Trust, Dialysis Unit
5. George Elliot Hospital NHS Trust, Nuneaton; Lucy Dean Dialysis Unit

Inclusion criteria: Participants will need to fulfill following criteria for enrolment in one of the three arms of the study:

1. On haemodialysis for at least 3months
2. On 3 times 4 hours of dialysis per week
3. Urea reduction rate of at least 65% during the three months before enrolment
4. Age 18 years or older

5. Able to complete the CPEX test and exercise training
6. Able to provide informed consent
7. Life expectancy of more than 6 months according to clinical assessment

Exclusion criteria:

1. Clinically significant valvular insufficiency
2. Clinically significant dysrythmia
3. Uncontrolled blood pressure: systolic > 160, diastolic >95 during the months before enrolment
4. Excessive fluid accumulation between dialysis sessions (>3 liters), more than twice pulmonary edema over 3 months before enrolment deemed to be due to excess fluid intake
5. Haemoglobin unstable and below 9.0
6. Ischemic cardiac event or intervention in the last 3 months
7. Morbidly obese, mid-thigh circumference of more than 60cm (EMS straps limit)
8. Clinically significant, still active inflammatory or malignant process
9. Pacemaker or cardiac device (contraindicated for bioelectrical impedance)
10. Patient highly physically active on their own accord.
11. Planned kidney transplant during study period.

Study design

This study is an open labeled, randomised controlled trial. Patients will be pre-screened using the clinical information available on their dialysis documentation. Eligible patients will be approached, patient information provided and then informed consent obtained. After randomisation patients will be allocated to one of the three study groups outlined in figure 1 below, with 12 weeks of dynamic exercise, LF-EMS or usual care.

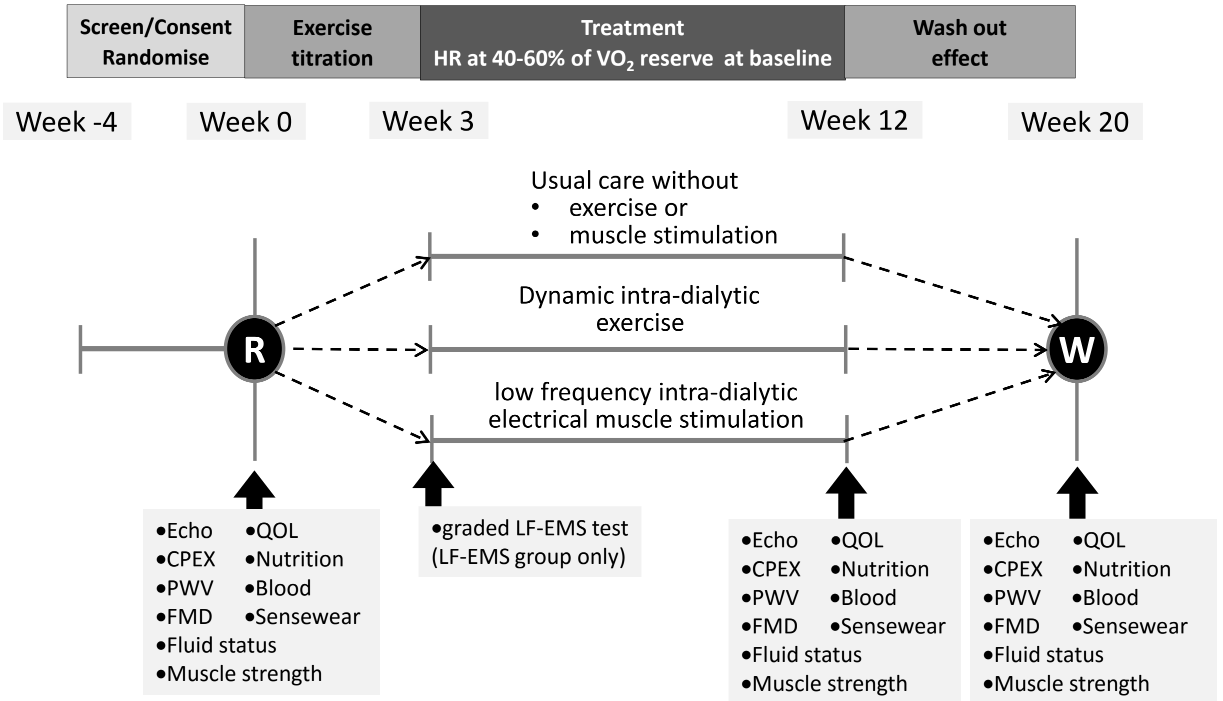

Figure 1: Study flow diagram – R= randomization, W= washout effect, CPEX = cardiopulmonary exercise test, PWV = pulse wave velocity, FMD = flow mediated dilation, QOL = quality of life

Study flow: After patients have been approached and consented, the duration of their participation will be 20-24 weeks, undergoing 20 weeks study specific procedures.

Consenting (week -3 to-1): Eligible patients will be approached by the research nurse or the investigator and information provided. At the second visit, while on dialysis, the patients will be consented and all the potential procedures explained again. This will include a video explaining the two modalities of exercise, dynamic and LF-EMS.

Baseline visit (week -1): This will coincide with a dialysis day and include obtaining clinical and QOL data, medication list, and a 'Sensewear' physical activity monitor armband will be fitted. 'Senswear' will allow validated measurement of free living physical activity over a 7 day period[70, 71].

Randomisation visit (week 0): This visit will occur on a non-dialysis day, during a short inter-dialytic interval and at least 12 hours after the last dialysis session. The visit will include an echocardiogram, bioelectrical impedance, CPEX with lung function test, applanation tonometry, flow mediated vascular dilatation, measurement of muscle strength and blood sampling for routine tests and for future testing of novel serum markers. Outcomes assessors will be blinded to the group allocation of the patient.

Participants will be randomised to one of 3 groups: 1) the intra-dialytic LF-EMS group, 2) the dynamic intra-dialytic exercise group or 3) the usual care without any dynamic exercise or LF-EMS group.

Treatment – familiarisation period (week 0-3)**:** Exercise under close instruction and supervision will be commenced in patients randomised to either of the exercise groups. Over the initial 3 weeks, LF-EMS and dynamic exercise duration will be gradually increased to 30 mins per dialysis session. Previous work has indicated that 3 weeks of regular LF-EMS exposure is required to achieve an intensity that will elicit a cardiovascular training stimulus [72] (i.e. 40-60% VO_2 reserve[73]). LF-EMS intensity will be gradually increased, as tolerated, with the aim of achieving the appropriate level by week 3. Dynamic exercise work load will be gradually increased to achieve a heart rate and workload equivalent to that achieved at 40-60% VO_2 reserve during baseline CPEX. As the use of heart rate during dialysis may not always provide an accurate reflection of workload[73], rating of perceived exertion (RPE) will also be used to monitor exercise intensity in both groups[72].

Graded LF-EMS test (week 3): to ensure that LF-EMS tolerance has improved to a level that elicits a workload of 40-60% VO_2 reserve, a graded LF-EMS test will be performed in this group following the 3 week familiarisation period. This will be performed during a dialysis session. Stimulation intensity will be incrementally increased every 5 minutes until the tolerable level is achieved[72] and oxygen consumption measured with the respiratory gas analysis system.

Treatment visit (weeks 3-12): Patients in the exercise groups will progress from 30-45 mins of exercise at an intensity of 40-60% VO_2 reserve as regulated by heart rate and RPE. Where this isn't possible, maximal tolerated exercise intensity will be continued.

Treatment completion visit (week 13): This visit will occur on a non-dialysis day, during a short inter-dialytic interval and at least 12 hours after last dialysis session. The visit will include recording of any clinical events, QOL assessment, free living with 'Senswear' for the following 7 days, echocardiogram, bioelectrical impedance, CPEX with lung function test, applanation tonometry, flow mediated vascular dilatation, measurement of muscle strength and blood sampling for routine tests and for future testing of novel serum markers. Outcomes assessors will be blinded to the group allocation of the patient.

Wash out and study completion visit (week 20): *This part of the study will only be performed, if further funding can be obtained.* This visit will occur on a non-dialysis day, during a short inter-dialytic interval and at least 12 hours after last dialysis session. The visit will include recording of any clinical events, QOL assessment and free living physical activity with 'Senswear' for the following 7 days, echocardiogram, bioelectrical impedance, CPEX with lung function test, applanation tonometry, flow mediated vascular dilatation, measurement of muscle strength and blood sampling for routine tests and for future testing of novel serum markers. During the 8 week 'washout' period, patients will undertake usual care which will not involve dynamic exercise training or LF-EMS.

Randomisation: Stratified permuted block randomisation with a variable block size (sizes 3 and 6 used) will be employed. Stratification variables are age (young, middle-aged, old) and gender (male, female), and a separate permuted block randomisation will be performed within each of the six strata. The randomisation list will be created and held by a Warwick Medical School statistician who the study team are going to contact to obtain the group allocation of a recruited patient. The randomisation list will not be made available to the study team.

Sample size calculation: Since this is a pilot study, no formal sample size calculation was conducted. The sample size was chosen based on Kieser and Wassmer (1996), who recommended recruiting 20 to 25 patients per group for a pilot study (with a normally distributed outcome) where the aim is to minimise the overall sample size of pilot and large scale RCT. Being conservative, a drop-out rate of 20% is assumed and thus a sample size of 30 patients per group has been chosen.

Dynamic exercise and electrical muscle stimulation treatment intervention

Low frequency electrical stimulation (LF-EMS) (treatment group): Bilateral neoprene straps will be applied to the quadriceps and hamstrings 3 times per week during the second hour of dialysis. Rhythmical contractions will occur at a frequency of 3-4 Hz. Participants will be introduced to the EMS protocol very gradually to ensure tolerance and adherence. On the first occasion the straps will be activated for 5 minutes at a very light current amplitude (milliamps, mA) (less than visible contraction), so that participants can become accustomed to the unusual sensation. During the following 8 familiarisation sessions (3 weeks), duration will be incrementally increased to 30 mins and intensity increased as tolerated. By the end of the third week the intention is that the patient will be accustomed to using the straps for 30 mins at an intensity equivalent to 40-60% VO2 reserve[72]. This will be assessed with an intra-dialytic graded LF-EMS CPEX (as above). During LF-EMS sessions, exercise intensity will be regulated using a combination of heart rate and rating of perceived exertion (RPE). Intensity will be further increased over the 9 week period of the intervention to the maximum tolerated level. This is necessary to ensure that, whilst cardiovascular reserve and skeletal muscle function improve, intensity remains at a level that will elicit a cardiovascular exercise response. Duration will also be increased to 45 mins by the second week of the intervention and will remain at this level throughout. Each session will be preceded by a 10 minute 'ramp-up' period during which intensity will be gradually increased to the training level[72]. Each session will be concluded with a 5 minute cool-down at a gradually reducing intensity. The LF-EMS units will record the intensity and duration of the stimulation which can be downloaded for analysis.

Dynamic exercise (exercise control group): Dynamic exercise will be performed 3 times per week during the second hour of dialysis using a recumbent exercise bike, or alternatively a leg ergometer whilst seated in a dialysis chair. On the first occasion, patients will exercise for 10-15 mins at an intensity with which they feel comfortable. During the following 8 familiarisation sessions (3 weeks) exercise duration will be

gradually increased to 30 mins. Intensity will be simultaneously increased until patients are capable of achieving 40-60% VO_2 reserve, as determined from CPEX. Intensity will be regulated and monitored using a combination of heart rate and rating of perceived exertion (RPE). Intensity will be periodically increased, as tolerated, over the 9 week period of the intervention. This is necessary to ensure that, whilst cardiovascular reserve increases and skeletal muscle function improves, intensity remains at a level that will elicit a cardiovascular exercise response. Duration will also be increased to 45 mins by the second week of the intervention and will remain at this level throughout. Each session will be preceded by a 5 minute warm-up period during which intensity will be gradually increased to the training level. Each session will be concluded with a 5 min cool down at a gradually reducing intensity. The intensity and duration of exercise performed at each session will be recorded.

LF-EMS Dynamic exercise

Usual care (no exercise control group): Participants randomised into this group will be supported, but will not receive any intra-dialytic exercise. They will also not be encouraged to participate in inter-dialytic exercise on their own. The amount of exercise will be determined with a questionnaire and with measurement of free living physical activity over a 7 day period ('Senswear') before randomisation, at the end of the intervention period (week 12) and at end of study (week 20).

Study outcome

Study aim 1: Tolerability of intra-dialytic dynamic exercise and LF-EMS interventions compared to conventional treatment without exercise intervention.

Study aim 2: Improvement of quality of life with intra-dialytic dynamic exercise and LF-EMS interventions compared to conventional treatment without exercise intervention .

***Study aim 3*:** Effects of intra-dialytic dynamic exercise training compared to conventional treatment without exercise intervention on measures of functional cardiovascular and skeletal muscle function.

Study aim 4: Effects of LF-EMS intra-dialytic exercise training compared to conventional treatment without exercise intervention on measures of functional cardiovascular and skeletal muscle function.

Study aim 5: Qualitatively compare the effects of LF-EMS with dynamic intra-dialytic exercise training on measures of functional cardiovascular and skeletal muscle function.

Outcome measures

The **primary outcome** measure is the tolerability, safety and acceptance of LF-EMS, compliance/ adherence to LF-EMS and improvement of QOL measures with LF-EMS in comparison to dynamic exercise and a conventional treatment approach.

The **secondary outcome** measures are cardiovascular and skeletal muscle improvement with LF-EMS in comparison to dynamic exercise and a conventional treatment approach.

Qualitative

Acceptance and tolerability: This will be assessed by recruitment rate into the study (target = 20% of the available population recruited (i.e. 90 out of 450) and recruitment targets met in the specified timescale of 18 months), willingness of participants to be randomised to LF-EMS, dynamic exercise or control; tolerance to the two interventions and compliance/adherence to the protocol (no more than 20% study dropout; reasons will be recorded).

Quality of life (QoL): Health related quality of life will be assessed using the Kidney Disease Quality of Life (KDQOL-36^{TM}) questionnaire which has been extensively validated in CKD populations[74, 75]. In addition, the Kansas City Cardiomyopathy Questionnaire will be administered which has been validated for use in stable and decompensated heart failure populations[76].

Nutritional status: To provide a global assessment of nutritional status, the Malnutrition-Inflammation Score[77] will be calculated following assessment by a renal dietitian, within 20 minutes of completion of a dialysis session. This tool is a validated measure of nutritional status in ESRD and is predictive of mortality in haemodialysis patients[78, 79].

Safety of intervention: During and following each session of LF-EMS or dynamic exercise, clinical parameters will be recorded (HR, BP, O_2 saturation) and the patient will be monitored for visible signs of clinical instability. Any adverse events will be recorded. The same data will be recorded in the usual care group to allow comparison.

Quantitative Cardiovascular

***Free living physical activity*:** The 'Senswear' physical activity monitor will allow validated measurement of free living physical activity over a 7 day period[70, 71]. This device, a small armband worn on the upper arm, measures motion with a tri-axial accelerometer, galvanic skin response (electrical conductivity of the skin, which changes in response to sweat and emotional stimuli), skin temperature and heat flux (amount of heat dissipating from the body). The following parameters will be recorded: total energy expenditure (kcal/min), active energy expenditure (kcal/min), METS (metabolic equivalent of activity), total number of steps, physical activity levels and duration, sleep duration and efficiency, lying down time, On/Off body time. Currently, there is no literature examining physical activity participation, assessed with this technique, in the CKD population.

CPEX: CPEX is an increasingly established tool in the medical management of CHF patients[80-82]. The clinical significance of this technology is demonstrated in its ability to quantify physical functional

capabilities, establish the severity of CHF and stratify risk of early death among these patients. CPEX-derived measures of maximal aerobic capacity (VO_2max) reflect the degree of ventricular function (pumping capacity), vascular function (O_2 delivery) and skeletal muscle metabolic capacity (O_2 utilization). This test will be carried out on the first non-dialysis day, at least 12 hours after the last dialysis session in order to avoid the effects of interdialytic weight gains and hemodialysis-induced myocardial stunning[83, 84]. Tests will be performed in accordance with American Thoracic Society guidelines [85]. A calibrated, electronically braked, upright exercise bicycle will be used in combination with an exercise respiratory gas analysis system. Gas analyser and volume calibrations will be undertaken daily and prior to each test respectively, as dictated by manufacturer's guidelines. Breath by breath respiratory gas exchange measurements of oxygen uptake (VO_2), carbon dioxide production (VCO_2) and minute ventilation (V_E) will be recorded and used to derive ventilatory threshold (VT) and ventilatory efficiency (VE/VCO_2 slope) [86]. The 'V slope' method and/or the dual criteria [87, 88] will be utilised to identify VT and the VE/VCO_2 slope will be measured from the start of unloaded pedalling to maximal exercise and calculated using linear regression. Commonly, a true $VO_{2\ max}$ (as determined by a plateau in VO_2) is not achieved in cardiovascular disease populations and thus the measure of peak VO_2 ($VO_{2\ peak}$) is preferred [89, 90]. Peak VO_2 will be determined in the last 30 seconds of exercise as the highest measured average of the mid five of every seven breaths. In addition to gas exchange variables, a twelve lead ECG will be continuously monitored and blood pressure and rating of perceived exertion (RPE) recorded every two minutes. A standard ramp protocol will be employed with increments of 10, 15, 20 or 25 W/min, calculated to ensure an optimal test duration of 9-12 minutes [90]. Three minutes of rest, followed by three minutes of unloaded pedalling, will precede the test and subjects will be strongly encouraged to maintain a cadence of 70 rpm until symptom limited volitional fatigue. In accordance with published criteria, a respiratory exchange ratio (RER) of >1.15 will be considered indicative of maximal effort [89].

***Echocardiography with Tissue Doppler (TDI) and Strain Imaging*:** Echocardiography will allow the assessment of cardiac structure and function using traditional and novel analysis techniques. Standard M-mode, 2-dimensional echocardiography, and Doppler blood flow measurements will be performed[91]. Calculations will include LV diastolic and systolic volumes, mass, ejection fraction (LVEF) according to Simpson's method and isovolumic relaxation time. Mitral inflow measurements will include peak early (E), peak late (A) flow velocities and the E/A ratio. The TDI of the mitral annulus will be obtained from the apical 4-chamber view. A 1.5 sample will be placed sequentially at the lateral and septal annular sites. Analysis will be performed for the early (E') diastolic peak velocity. The ratio of early transmitral flow velocity to annular mitral velocity of the lateral LV wall (E/E') will be taken as an estimate of LV filling pressure. Myocardial deformation (strain and strain rate) will be measured in three planes (longitudinal, circumferential and radial) in the apical 4-chamber view, and the parasternal short axis views at the base (mitral valve) and apex using 2D speckle tracking[92].

Bioelectrical impedance: Fluid volume status and body composition will be assessed with direct segmental multi-frequency bioelectrical impedance analysis (Biospace Inbody 720). This is currently the recommended method for assessment of fluid status in haemodialysis patients[93]. The primary aim of this assessment is to ensure that fluid volume is comparable at the baseline testing visit and the study completion visit, thus minimising the impact of fluid volume on the assessment of cardiac and vascular function. Patients will stand on the bioelectrical impedance machine whilst eight electrodes are attached to the body.

Arterial applanation tonometry: Resting brachial blood pressure will be measured following 10 minutes of supine rest. Pulse wave analysis will be performed on the radial artery using a high fidelity micromanometer in accordance with a validated protocol[94]. Aortic pulse wave-form, augmentation

pressure, augmentation index (AIx) and time to reflection (Tr) will be derived using a validated radial-to-aortic transfer function (SphygmoCor). The systolic part of the aortic wave-form is characterised by the incident LV ejection pressure peak followed by the second pressure peak caused by the reflected wave. The augmented pressure is the difference between these two peak pressures and the ratio of the augmented pressure to pulse pressure (=systolic minus diastolic blood pressures) defines the AIx. As AIx is influenced by heart rate, an index adjusted to a heart rate of 75 beats/minute (AIx_{75}) will be recorded[95]. Tr is a measure of time of the return of the reflected waves to the central circulation. Using a similar technique, aortic (carotid-femoral) pulse wave velocity (PWV) will be determined by sequential recording of ECG-gated carotid and femoral waveforms.

Functional vascular assessment: Endothelial function will be assessed using flow-mediated dilation (FMD) of the non-fistula arm brachial artery[96]. Baseline artery blood flow and diameter will be measured using ultrasound. A blood pressure cuff will be inflated to >200 mmHg for five minutes. After rapid cuff release, brachial artery ultrasound will be repeated to demonstrate hyperaemia. Arterial diameter will be measured continuously for three minutes following cuff deflation, with maximal diameter being recorded. FMD is expressed as the percentage change in artery diameter after cuff deflation, compared to the pre-inflation measurement.

Quantitative skeletal muscle

Muscle strength: Leg strength will be measured using a validated hand held dynamometry technique[97]. Subjects will sit in an elevated chair with foam padding and stabilisation straps for the waist and thigh. The subject will attempt to straighten their leg and perform 3 maximal contractions (with 30 seconds rest between each) against a dynamometer which will measure the force of contraction.

Serum markers

Blood sample: Routine testing will include Full blood cell count, Reticulocytes, Iron parameters (Iron, Transferrin, Saturation), Renal function /dialysis adequacy (Creatinine, Urea, Sodium, Potassium), Bone metabolism (corrected Calcium, Phosphate, Alkaline phosphatase, Alkaline phosphatase, 25-hydroxyvitamin D), Liver function (Total protein, Albumin, Bilirubin, ALT), inflammation (CRP), glycemic control (HbA1C), lipids.

Serum and plasma will be stored for further analysis:

1. **Cardiac stress: NT-pro-BNP;** NT-pro-BNP is representative of LV wall stress and thus indicative of LV hemodynamic compromise [98]. Raised NT-pro-BNP is incrementally associated with poor prognosis and adverse outcomes in CHF[99]. The independent increase in B-type natriuretic peptide (BNP) in response to increasing LV mass index has been observed in CKD patients[100]. With exercise training in CHF, meta-analyses have reported reductions in NT-pro-BNP (37%), indicating improved LV hemodynamics[101]. This has not been studied in CKD. **Troponin T (cTnT):** raised cTnT demonstrates a strong association with pathologic LV hypertrophy in CKD[102] and is independently associated with cardiovascular events and mortality[103].
2. **Inflammation: hsCRP, Calprotectin, Cathelicidin;** inflammation is one of the main markers of poor outcome in CKD. CRP is associated with all-cause and CV mortality in ESRD[104]. Novel

inflammatory biomarkers such as Calprotectin and Cathelicidin represent potential future indicators of an improved inflammatory environment with exercise training in CKD.

3. **Protein catabolism: Gelsolin,** an actin-binding protein that is a key regulator of actin filament assembly and disassembly**,** and **Myostatin,** a negative regulator of skeletal muscle mass, are novel markers of hypercatabolism in CKD. Limited data suggests an exercise induced reduction in myostatin in CHF and obesity[105, 106], whereas the effects of exercise on Gelsolin have not been studied.
4. **Iron metabolism: Iron, Transferrin and saturation, Hepcidin**, an acute phase reactant protein and a key regulator of iron homeostasis is increased with inflammation in CKD, limiting the availability of iron, thus contributing to anaemia[107]. The potential for improved iron metabolism through an exercise induced reduction in hepcidin has not been explored in the CKD population.

Statistical analyses

The two primary objectives of this study are to generate estimates, in particular variance estimates, for a sample size calculation of a subsequent fully powered RCT and to determine the likelihood of a successful conduct of such an RCT. The latter will be assessed by estimating recruitment and drop-out rate indication feasibility of a large-scale trial. Since this study is a pilot the statistical analysis will be primarily descriptive. Point estimates and corresponding 95% confidence intervals will be calculated for all outcome measures in the three arms and the aforementioned rates. Furthermore the distribution of outcome measures will be assessed to identify appropriate statistical analysis methods for a future RCT. Between group comparisons via statistical tests (unpaired two-sample t-test for continuous outcomes, Chi-squared tests for categorical (binary) outcomes) will be conducted, however, it will be clearly stated that these yield only preliminary results since the study is not powered for such investigations. The results of these tests will only be used to identify overwhelming evidence of inferiority of the two experimental treatments relative to usual care so that plans of a full trial can be abandoned or an arm dropped. In accordance with Kieser and Wassmer (1996), the upper 90% confidence limit will be chosen as the estimate of the variance of the treatment effect for a future trial's sample size calculation. Intention-to-treat and per protocol analyses will both be conducted to determine potential ranges of results.

References:

1. NICE. Guideline CG73 Chronic KIdney Disease.
2. Stevens PE, O'Donoghue DJ, de Lusignan S, et al. Chronic kidney disease management in the United Kingdom: NEOERICA project results. *Kidney Int*. 2007; 72: 92-9.
3. System URD. USRD 2006 annual data report Bethesda, MD: National Institutes of Health, National Institute of Diabetes and Digestive and Kidney Diseases, 2006.
4. Fox CS, Matsushita K, Woodward M, et al. Associations of kidney disease measures with mortality and end-stage renal disease in individuals with and without diabetes: a meta-analysis. *Lancet*. 2012; 380: 1662-73.
5. Mahmoodi BK, Matsushita K, Woodward M, et al. Associations of kidney disease measures with mortality and end-stage renal disease in individuals with and without hypertension: a meta-analysis. *Lancet*. 2012; 380: 1649-61.
6. Hostetter TH. Chronic kidney disease predicts cardiovascular disease. *N Engl J Med*. 2004; 351: 1344-6.
7. Drey N, Roderick P, Mullee M and Rogerson M. A population-based study of the incidence and outcomes of diagnosed chronic kidney disease. *Am J Kidney Dis*. 2003; 42: 677-84.
8. Trespalacios FC, Taylor AJ, Agodoa LY, Bakris GL and Abbott KC. Heart failure as a cause for hospitalization in chronic dialysis patients. *Am J Kidney Dis*. 2003; 41: 1267-77.
9. Gansevoort RT, Correa-Rotter R, Hemmelgarn BR, et al. Chronic kidney disease and cardiovascular risk: epidemiology, mechanisms, and prevention. *Lancet*. 2013; 382: 339-52.
10. Coca SG, Krumholz HM, Garg AX and Parikh CR. Underrepresentation of renal disease in randomized controlled trials of cardiovascular disease. *JAMA*. 2006; 296: 1377-84.
11. Davies EJ, Moxham T, Rees K, et al. Exercise training for systolic heart failure: Cochrane systematic review and meta-analysis. *Eur J Heart Fail*. 2010; 12: 706-15.
12. Heiwe S and Jacobson SH. Exercise training for adults with chronic kidney disease. *Cochrane Database Syst Rev*. 2011: CD003236.
13. Voet NB, van der Kooi EL, Riphagen, II, Lindeman E, van Engelen BG and Geurts AC. Strength training and aerobic exercise training for muscle disease. *Cochrane Database Syst Rev*. 2013; 7: CD003907.
14. Heran BS, Chen JM, Ebrahim S, et al. Exercise-based cardiac rehabilitation for coronary heart disease. *Cochrane Database Syst Rev*. 2011: CD001800.
15. Lee BC, Chen SY, Hsu HC, et al. Effect of cardiac rehabilitation on myocardial perfusion reserve in postinfarction patients. *Am J Cardiol*. 2008; 101: 1395-402.
16. Giallauria F, Cirillo P, Lucci R, et al. Left ventricular remodelling in patients with moderate systolic dysfunction after myocardial infarction: favourable effects of exercise training and predictive role of N-terminal pro-brain natriuretic peptide. *Eur J Cardiovasc Prev Rehabil*. 2008; 15: 113-8.
17. Zeng S, Zhou QC, Peng QH, et al. Assessment of regional myocardial function in patients with dilated cardiomyopathy by velocity vector imaging. *Echocardiography*. 2009; 26: 163-70.
18. Valkeinen H, Aaltonen S and Kujala UM. Effects of exercise training on oxygen uptake in coronary heart disease: a systematic review and meta-analysis. *Scand J Med Sci Sports*. 2010; 20: 545-55.
19. Lawler PR, Filion KB and Eisenberg MJ. Efficacy of exercise-based cardiac rehabilitation post-myocardial infarction: a systematic review and meta-analysis of randomized controlled trials. *Am Heart J*. 2011; 162: 571-84 e2.
20. Gielen S, Schuler G and Adams V. Cardiovascular effects of exercise training: molecular mechanisms. *Circulation*. 2010; 122: 1221-38.
21. Kemi OJ and Wisloff U. Mechanisms of exercise-induced improvements in the contractile apparatus of the mammalian myocardium. *Acta Physiol (Oxf)*. 2010; 199: 425-39.
22. Schober T and Knollmann BC. Exercise after myocardial infarction improves contractility and decreases myofilament Ca2+ sensitivity. *Circ Res*. 2007; 100: 937-9.
23. Kemi OJ, Ellingsen O, Ceci M, et al. Aerobic interval training enhances cardiomyocyte contractility and Ca2+ cycling by phosphorylation of CaMKII and Thr-17 of phospholamban. *J Mol Cell Cardiol*. 2007; 43: 354-61.
24. McMullen JR, Amirahmadi F, Woodcock EA, et al. Protective effects of exercise and phosphoinositide 3-kinase(p110alpha) signaling in dilated and hypertrophic cardiomyopathy. *Proc Natl Acad Sci U S A*. 2007; 104: 612-7.
25. Haykowsky M, Scott J, Esch B, et al. A meta-analysis of the effects of exercise training on left ventricular remodeling following myocardial infarction: start early and go longer for greatest exercise benefits on remodeling. *Trials*. 2011; 12: 92.
26. Chen YM, Li ZB, Zhu M and Cao YM. Effects of exercise training on left ventricular remodelling in heart failure patients: an updated meta-analysis of randomised controlled trials. *Int J Clin Pract*. 2012; 66: 782-91.
27. Aoki J, Ikari Y, Nakajima H, et al. Clinical and pathologic characteristics of dilated cardiomyopathy in hemodialysis patients. *Kidney Int*. 2005; 67: 333-40.
28. Raggi P, Boulay A, Chasan-Taber S, et al. Cardiac calcification in adult hemodialysis patients. A link between end-stage renal disease and cardiovascular disease? *J Am Coll Cardiol*. 2002; 39: 695-701.

29. Perkovic V, Hunt D, Griffin SV, du Plessis M and Becker GJ. Accelerated progression of calcific aortic stenosis in dialysis patients. *Nephron Clin Pract*. 2003; 94: c40-5.
30. Cerasola G, Nardi E, Palermo A, Mule G and Cottone S. Epidemiology and pathophysiology of left ventricular abnormalities in chronic kidney disease: a review. *J Nephrol*. 2011; 24: 1-10.
31. Guyton AC and Coleman TG. Quantitative analysis of the pathophysiology of hypertension. 1969. *J Am Soc Nephrol*. 1999; 10: 2248-58.
32. Tsioufis C, Vezali E, Tsiachris D, et al. Left ventricular hypertrophy versus chronic kidney disease as predictors of cardiovascular events in hypertension: a Greek 6-year-follow-up study. *J Hypertens*. 2009; 27: 744-52.
33. Green D, Roberts PR, New DI and Kalra PA. Sudden cardiac death in hemodialysis patients: an in-depth review. *Am J Kidney Dis*. 2011; 57: 921-9.
34. (USRDS) URDS. Annual Data Report: Atlas of Chronic Kidney Disease and End-Stage Renal Disease in the United States In: National Institutes of Health NIoDaDaKD, (ed.). Bethesda, MD2011.
35. Ganesh SK, Stack AG, Levin NW, Hulbert-Shearon T and Port FK. Association of elevated serum PO(4), Ca x PO(4) product, and parathyroid hormone with cardiac mortality risk in chronic hemodialysis patients. *J Am Soc Nephrol*. 2001; 12: 2131-8.
36. Ronco C, Haapio M, House AA, Anavekar N and Bellomo R. Cardiorenal syndrome. *J Am Coll Cardiol*. 2008; 52: 1527-39.
37. Edwards NC, Steeds RP, Ferro CJ and Townend JN. The treatment of coronary artery disease in patients with chronic kidney disease. *QJM*. 2006; 99: 723-36.
38. Blacher J, Pannier B, Guerin AP, Marchais SJ, Safar ME and London GM. Carotid arterial stiffness as a predictor of cardiovascular and all-cause mortality in end-stage renal disease. *Hypertension*. 1998; 32: 570-4.
39. O'Rourke MF and Safar ME. Relationship between aortic stiffening and microvascular disease in brain and kidney: cause and logic of therapy. *Hypertension*. 2005; 46: 200-4.
40. O'Rourke MF. Arterial pressure waveforms in hypertension. *Minerva Med*. 2003; 94: 229-50.
41. London GM. Left ventricular hypertrophy: why does it happen? *Nephrol Dial Transplant*. 2003; 18 Suppl 8: viii2-6.
42. Edwards NC, Ferro CJ, Townend JN and Steeds RP. Aortic distensibility and arterial-ventricular coupling in early chronic kidney disease: a pattern resembling heart failure with preserved ejection fraction. *Heart*. 2008; 94: 1038-43.
43. Gross ML and Ritz E. Hypertrophy and fibrosis in the cardiomyopathy of uremia--beyond coronary heart disease. *Semin Dial*. 2008; 21: 308-18.
44. Moody WE, Edwards NC, Chue CD, Ferro CJ and Townend JN. Arterial disease in chronic kidney disease. *Heart*. 2013; 99: 365-72.
45. Briet M and Burns KD. Chronic kidney disease and vascular remodelling: molecular mechanisms and clinical implications. *Clin Sci (Lond)*. 2012; 123: 399-416.
46. Morris ST, McMurray JJ, Spiers A and Jardine AG. Impaired endothelial function in isolated human uremic resistance arteries. *Kidney Int*. 2001; 60: 1077-82.
47. Verbeke FH, Pannier B, Guerin AP, Boutouyrie P, Laurent S and London GM. Flow-mediated vasodilation in end-stage renal disease. *Clin J Am Soc Nephrol*. 2011; 6: 2009-15.
48. Stam F, van Guldener C, Becker A, et al. Endothelial dysfunction contributes to renal function-associated cardiovascular mortality in a population with mild renal insufficiency: the Hoorn study. *J Am Soc Nephrol*. 2006; 17: 537-45.
49. Fujihara CK, De Nucci G and Zatz R. Chronic nitric oxide synthase inhibition aggravates glomerular injury in rats with subtotal nephrectomy. *J Am Soc Nephrol*. 1995; 5: 1498-507.
50. Desmeules S, Levesque R, Jaussent I, Leray-Moragues H, Chalabi L and Canaud B. Creatinine index and lean body mass are excellent predictors of long-term survival in haemodiafiltration patients. *Nephrol Dial Transplant*. 2004; 19: 1182-9.
51. Lenk K, Schuler G and Adams V. Skeletal muscle wasting in cachexia and sarcopenia: molecular pathophysiology and impact of exercise training. *J Cachexia Sarcopenia Muscle*. 2010; 1: 9-21.
52. Raj DS, Sun Y and Tzamaloukas AH. Hypercatabolism in dialysis patients. *Curr Opin Nephrol Hypertens*. 2008; 17: 589-94.
53. Deligiannis A, Kouidi E, Tassoulas E, Gigis P, Tourkantonis A and Coats A. Cardiac effects of exercise rehabilitation in hemodialysis patients. *Int J Cardiol*. 1999; 70: 253-66.
54. Koufaki P, Mercer TH and Naish PF. Effects of exercise training on aerobic and functional capacity of end-stage renal disease patients. *Clin Physiol Funct Imaging*. 2002; 22: 115-24.
55. Kouidi EJ, Grekas DM and Deligiannis AP. Effects of exercise training on noninvasive cardiac measures in patients undergoing long-term hemodialysis: a randomized controlled trial. *Am J Kidney Dis*. 2009; 54: 511-21.
56. Chen JL, Godfrey S, Ng TT, et al. Effect of intra-dialytic, low-intensity strength training on functional capacity in adult haemodialysis patients: a randomized pilot trial. *Nephrol Dial Transplant*. 2010; 25: 1936-43.

57. Makhlough A, Ilali E, Mohseni R and Shahmohammadi S. Effect of intradialytic aerobic exercise on serum electrolytes levels in hemodialysis patients. *Iran J Kidney Dis*. 2012; 6: 119-23.
58. Cheema B, Abas H, Smith B, et al. Randomized controlled trial of intradialytic resistance training to target muscle wasting in ESRD: the Progressive Exercise for Anabolism in Kidney Disease (PEAK) study. *Am J Kidney Dis*. 2007; 50: 574-84.
59. Toussaint ND, Polkinghorne KR and Kerr PG. Impact of intradialytic exercise on arterial compliance and B-type natriuretic peptide levels in hemodialysis patients. *Hemodial Int*. 2008; 12: 254-63.
60. Segura-Orti E. [Exercise in haemodyalisis patients: a literature systematic review]. *Nefrologia*. 2010; 30: 236-46.
61. Smart NA, Dieberg G and Giallauria F. Functional electrical stimulation for chronic heart failure: a meta-analysis. *Int J Cardiol*. 2012; 167: 80-6.
62. Dobsak P, Novakova M, Fiser B, et al. Electrical stimulation of skeletal muscles. An alternative to aerobic exercise training in patients with chronic heart failure? *Int Heart J*. 2006; 47: 441-53.
63. Dobsak P, Novakova M, Siegelova J, et al. Low-frequency electrical stimulation increases muscle strength and improves blood supply in patients with chronic heart failure. *Circ J*. 2006; 70: 75-82.
64. Bourjeily-Habr G, Rochester CL, Palermo F, Snyder P and Mohsenin V. Randomised controlled trial of transcutaneous electrical muscle stimulation of the lower extremities in patients with chronic obstructive pulmonary disease. *Thorax*. 2002; 57: 1045-9.
65. Grosset JF, Crowe L, De Vito G, O'Shea D and Caulfield B. Comparative effect of a 1 h session of electrical muscle stimulation and walking activity on energy expenditure and substrate oxidation in obese subjects. *Appl Physiol Nutr Metab*. 2013; 38: 57-65.
66. Banerjee P, Caulfield B, Crowe L and Clark A. Prolonged electrical muscle stimulation exercise improves strength and aerobic capacity in healthy sedentary adults. *J Appl Physiol (1985)*. 2005; 99: 2307-11.
67. Banerjee P, Clark A, Witte K, Crowe L and Caulfield B. Electrical stimulation of unloaded muscles causes cardiovascular exercise by increasing oxygen demand. *Eur J Cardiovasc Prev Rehabil*. 2005; 12: 503-8.
68. Crognale D, Vito GD, Grosset JF, Crowe L, Minogue C and Caulfield B. Neuromuscular electrical stimulation can elicit aerobic exercise response without undue discomfort in healthy physically active adults. *J Strength Cond Res*. 2012; 27: 208-15.
69. Dobsak P, Tomandl J, Spinarova L, et al. Effects of neuromuscular electrical stimulation and aerobic exercise training on arterial stiffness and autonomic functions in patients with chronic heart failure. *Artif Organs*. 2012; 36: 920-30.
70. Brazeau AS, Karelis AD, Mignault D, Lacroix MJ, Prud'homme D and Rabasa-Lhoret R. Test-retest reliability of a portable monitor to assess energy expenditure. *Appl Physiol Nutr Metab*. 2011; 36: 339-43.
71. Casiraghi F, Lertwattanarak R, Luzi L, et al. Energy Expenditure Evaluation in Humans and Non-Human Primates by SenseWear Armband. Validation of Energy Expenditure Evaluation by SenseWear Armband by Direct Comparison with Indirect Calorimetry. *PLoS One*. 2013; 8: e73651.
72. Crognale D, Vito GD, Grosset JF, Crowe L, Minogue C and Caulfield B. Neuromuscular electrical stimulation can elicit aerobic exercise response without undue discomfort in healthy physically active adults. *J Strength Cond Res*. 2013; 27: 208-15.
73. ACSM. *Guidelines for exercise testing and prescription*. 8th ed. Riverwoods, IL: Lippincott Williams & Wilkins, 2009.
74. Hays RD, Kallich JD, Mapes DL, Coons SJ and Carter WB. Development of the kidney disease quality of life (KDQOL) instrument. *Qual Life Res*. 1994; 3: 329-38.
75. Korevaar JC, Merkus MP, Jansen MA, Dekker FW, Boeschoten EW and Krediet RT. Validation of the KDQOL-SF: a dialysis-targeted health measure. *Qual Life Res*. 2002; 11: 437-47.
76. Green CP, Porter CB, Bresnahan DR and Spertus JA. Development and evaluation of the Kansas City Cardiomyopathy Questionnaire: a new health status measure for heart failure. *J Am Coll Cardiol*. 2000; 35: 1245-55.
77. Kalantar-Zadeh K, Kopple JD, Block G and Humphreys MH. A malnutrition-inflammation score is correlated with morbidity and mortality in maintenance hemodialysis patients. *Am J Kidney Dis*. 2001; 38: 1251-63.
78. Rambod M, Bross R, Zitterkoph J, et al. Association of Malnutrition-Inflammation Score with quality of life and mortality in hemodialysis patients: a 5-year prospective cohort study. *Am J Kidney Dis*. 2009; 53: 298-309.
79. Rambod M, Kovesdy CP and Kalantar-Zadeh K. Malnutrition-Inflammation Score for risk stratification of patients with CKD: is it the promised gold standard? *Nat Clin Pract Nephrol*. 2008; 4: 354-5.
80. Mancini DM, Eisen H, Kussmaul W, Mull R, Edmunds LH, Jr. and Wilson JR. Value of peak exercise oxygen consumption for optimal timing of cardiac transplantation in ambulatory patients with heart failure. *Circulation*. 1991; 83: 778-86.
81. ATS/ACCP Statement on cardiopulmonary exercise testing. *Am J Respir Crit Care Med*. 2003; 167: 211-77.
82. Hunt SA. ACC/AHA 2005 guideline update for the diagnosis and management of chronic heart failure in the adult: a report of the American College of Cardiology/American Heart Association Task Force on Practice

Guidelines (Writing Committee to Update the 2001 Guidelines for the Evaluation and Management of Heart Failure). *J Am Coll Cardiol*. 2005; 46: e1-82.
83. Burton JO, Jefferies HJ, Selby NM and McIntyre CW. Hemodialysis-induced repetitive myocardial injury results in global and segmental reduction in systolic cardiac function. *Clin J Am Soc Nephrol*. 2009; 4: 1925-31.
84. Burton JO, Jefferies HJ, Selby NM and McIntyre CW. Hemodialysis-induced cardiac injury: determinants and associated outcomes. *Clin J Am Soc Nephrol*. 2009; 4: 914-20.
85. Ross RM. ATS/ACCP statement on cardiopulmonary exercise testing. *Am J Respir Crit Care Med*. 2003; 167: 1451; author reply
86. Guazzi M, Adams V, Conraads V, et al. EACPR/AHA Scientific Statement. Clinical recommendations for cardiopulmonary exercise testing data assessment in specific patient populations. *Circulation*. 2012; 126: 2261-74.
87. Beaver WL, Wasserman K and Whipp BJ. A new method for detecting anaerobic threshold by gas exchange. *J Appl Physiol*. 1986; 60: 2020-7.
88. Wasserman K, Whipp BJ and Davis JA. Respiratory physiology of exercise: metabolism, gas exchange, and ventilatory control. *Int Rev Physiol*. 1981; 23: 149-211.
89. Balady GJ, Arena R, Sietsema K, et al. Clinician's Guide to cardiopulmonary exercise testing in adults: a scientific statement from the American Heart Association. *Circulation*. 2010; 122: 191-225.
90. Cooper C and Storer T. *Exercise testing and interpretation: a practical approach*. Cambridge, UK: Cambridge University Press, 2006.
91. Cheitlin MD, Armstrong WF, Aurigemma GP, et al. ACC/AHA/ASE 2003 Guideline Update for the Clinical Application of Echocardiography: summary article. A report of the American College of Cardiology/American Heart Association Task Force on Practice Guidelines (ACC/AHA/ASE Committee to Update the 1997 Guidelines for the Clinical Application of Echocardiography). *J Am Soc Echocardiogr*. 2003; 16: 1091-110.
92. Hoit BD. Strain and strain rate echocardiography and coronary artery disease. *Circ Cardiovasc Imaging*. 2011; 4: 179-90.
93. Dou Y, Zhu F and Kotanko P. Assessment of extracellular fluid volume and fluid status in hemodialysis patients: current status and technical advances. *Semin Dial*. 2012; 25: 377-87.
94. Tomlinson LA. Methods for assessing arterial stiffness: technical considerations. *Curr Opin Nephrol Hypertens*. 2012; 21: 655-60.
95. Wilkinson IB, Mohammad NH, Tyrrell S, et al. Heart rate dependency of pulse pressure amplification and arterial stiffness. *Am J Hypertens*. 2002; 15: 24-30.
96. Thijssen DH, Black MA, Pyke KE, et al. Assessment of flow-mediated dilation in humans: a methodological and physiological guideline. *Am J Physiol Heart Circ Physiol*. 2010; 300: H2-12.
97. Stark T, Walker B, Phillips JK, Fejer R and Beck R. Hand-held dynamometry correlation with the gold standard isokinetic dynamometry: a systematic review. *PM R*. 2011; 3: 472-9.
98. Ruskoaho H. Cardiac hormones as diagnostic tools in heart failure. *Endocr Rev*. 2003; 24: 341-56.
99. Masson S, Latini R, Anand IS, et al. Prognostic value of changes in N-terminal pro-brain natriuretic peptide in Val-HeFT (Valsartan Heart Failure Trial). *J Am Coll Cardiol*. 2008; 52: 997-1003.
100. Vickery S, Price CP, John RI, et al. B-type natriuretic peptide (BNP) and amino-terminal proBNP in patients with CKD: relationship to renal function and left ventricular hypertrophy. *Am J Kidney Dis*. 2005; 46: 610-20.
101. Smart NA, Meyer T, Butterfield JA, et al. Individual patient meta-analysis of exercise training effects on systemic brain natriuretic peptide expression in heart failure. *Eur J Prev Cardiol*. 2011; 19: 428-35.
102. Mishra RK, Li Y, DeFilippi C, et al. Association of cardiac troponin T with left ventricular structure and function in CKD. *Am J Kidney Dis*. 2013; 61: 701-9.
103. Dubin RF, Li Y, He J, et al. Predictors of high sensitivity cardiac troponin T in chronic kidney disease patients: a cross-sectional study in the chronic renal insufficiency cohort (CRIC). *BMC Nephrol*. 2013; 14: 229.
104. Menon V, Greene T, Wang X, et al. C-reactive protein and albumin as predictors of all-cause and cardiovascular mortality in chronic kidney disease. *Kidney Int*. 2005; 68: 766-72.
105. Lenk K, Erbs S, Hollriegel R, et al. Exercise training leads to a reduction of elevated myostatin levels in patients with chronic heart failure. *Eur J Prev Cardiol*. 2010; 19: 404-11.
106. Ryan AS, Li G, Blumenthal JB and Ortmeyer HK. Aerobic exercise + weight loss decreases skeletal muscle myostatin expression and improves insulin sensitivity in older adults. *Obesity (Silver Spring)*. 2013; 21: 1350-6.
107. Zaritsky J, Young B, Wang HJ, et al. Hepcidin--a potential novel biomarker for iron status in chronic kidney disease. *Clin J Am Soc Nephrol*. 2009; 4: 1051-6.
